# Non-targeted analysis of unexpected food contaminants using LC-HRMS

**DOI:** 10.1007/s00216-018-1028-4

**Published:** 2018-03-29

**Authors:** Marco Kunzelmann, Martin Winter, Magnus Åberg, Karl-Erik Hellenäs, Johan Rosén

**Affiliations:** 1 Chemistry Department, National Food Agency, Box 622, 751 26 Uppsala, Sweden; 2 Department of Environmental Science and Analytical Chemistry, Stockholm University, Svante Arrhenius väg 8, 114 18 Stockholm, Sweden

**Keywords:** Non-targeted analysis, Food contaminants, Food safety, HRMS, LC-MS, Unknown analysis

## Abstract

A non-target analysis method for unexpected contaminants in food is described. Many current methods referred to as “non-target” are capable of detecting hundreds or even thousands of contaminants. However, they will typically still miss all other possible contaminants. Instead, a metabolomics approach might be used to obtain “true non-target” analysis. In the present work, such a method was optimized for improved detection capability at low concentrations. The method was evaluated using 19 chemically diverse model compounds spiked into milk samples to mimic unknown contamination. Other milk samples were used as reference samples. All samples were analyzed with UHPLC-TOF-MS (ultra-high-performance liquid chromatography time-of-flight mass spectrometry), using reversed-phase chromatography and electrospray ionization in positive mode. Data evaluation was performed by the software TracMass 2. No target lists of specific compounds were used to search for the contaminants. Instead, the software was used to sort out all features only occurring in the spiked sample data, i.e., the workflow resembled a metabolomics approach. Procedures for chemical identification of peaks were outside the scope of the study. Method, study design, and settings in the software were optimized to minimize manual evaluation and faulty or irrelevant hits and to maximize hit rate of the spiked compounds. A practical detection limit was established at 25 μg/kg. At this concentration, most compounds (17 out of 19) were detected as intact precursor ions, as fragments or as adducts. Only 2 irrelevant hits, probably natural compounds, were obtained. Limitations and possible practical use of the approach are discussed.

**Electronic supplementary material:**

The online version of this article (10.1007/s00216-018-1028-4) contains supplementary material, which is available to authorized users.

## Introduction

Routine food safety analysis did not reveal the tainted milk scandal in China 2008 since melamine was not on the target list for control at that time [1]. As a consequence of the melamine adulteration, almost 300,000 babies were taken ill and 6 died as reported by the Ministry of Health, China, at the end of that year [2, 3]. More recent examples of unexpected food-related contamination of public concern are PFAS and related compounds in drinking water in several countries in Europe and the USA, e.g., in Sweden in 2012 [4, 5], and fipronil in Dutch eggs spread in Europe in 2017 [6]. Again, the compounds were unexpected and therefore not screened for until it was too late to prevent them from being widely spread, and some contaminated Swedish sources of raw water can still (year 2017) not be used for drinking water production.

While chemical measurements constitute one of the pillars of food safety, the current framework typically focuses only on substances that are legally regulated and expected to be found in specific foods. The risk is high that new or unexpected contamination due to fraud, mistakes, accidents etc. will not be revealed in time as exemplified above. Moreover, one might expect that the ongoing globalization makes the food supply chain more vulnerable to fraud as well as unintentional contamination, as exemplified by the fraudulent replacement of beef by low-quality horsemeat in ready-to-eat dishes like lasagna (Europe 2013) [7]. There is consequently a need for strategies to be able to act more proactively rather than, as in the examples, reactively.

One of the aims of today’s food safety paradigm is to minimize the risk for chronic effects from lifelong exposure of designated substances. Hence, these are routinely screened for at low levels, often in the micrograms per kilogram range, by target methods using sensitive and specific analytical techniques [8], typically based on GC-, or LC-MS/MS triple quadrupoles. The introduction of AM HRMS (accurate-mass high-resolution mass spectrometry) instruments suited for routine analysis has though led to new trends in food safety and environmental analysis, to combine target, suspect, and non-target screening, e.g., reviewed in 2015 for water analysis by Schymanski et al. [9]. Typical HRMS techniques referred to are TOF (time-of-flight) and Orbitrap, delivering data with comparable format and quality.

One benefit with MS/MS triple quadrupoles as compared to HRMS techniques is better sensitivity. However, if that is not needed, there are several benefits using the mentioned HRMS techniques. The data acquisition step is non-targeted and it is therefore possible to decide *after* the acquisition whether the generated data files should be used for (a) target, (b) suspect or (c) (true) non-target screening, or even a combination of all three approaches. (a) Target screening using HRMS typically involves a reference standard for obtaining information about retention time and fragmentation. (b) In suspect screening, a reference standard is not needed. Instead, the exact mass and isotopic pattern calculated from the molecular formula is used to screen for substances. Both these approaches—target and suspect screening using HRMS—are often referred to as non-target HRMS analysis since the data was acquired non-targeted. However, they are both targeted in the sense that they start with compound information followed by analyzing the MS data. (c) A (true) non-targeted screening approach is rather the opposite: it starts with the MS data and aims to reveal what is in the sample, or what is the difference between samples.

Such (true) non-target analysis by HRMS can be described as a two-step approach: (1) prioritization of signals, i.e., finding masses of interest, e.g., due to differences between samples and (2) identification of the compound behind these signals starting from the exact mass, isotope, adduct, and fragmentation information. The present article focuses on the prioritization step via a metabolomics approach. A similar study was earlier performed by our laboratory in order to develop a method and investigate its potential to aid prioritization of signals from unexpected contaminants in orange juice [10]. The latter study did not systematically investigate at how low concentrations the method could give meaningful results. The aim of the present study is to do so, this time—bearing the melamine scandal in mind—applied on milk.

## Materials and methods

Sample extraction, analysis, and data processing were based on an earlier study from the same laboratory as described in 2013 by Tengstrand et al. [10].

### Sample set-up

Milk samples of three labels were bought from a local supermarket (see Table 1). One label (A) was used for spiking, and the other two (B and C)—each with three different fat concentrations (3.0, 1.5, and 0.5%)—were used for reference. From each sample, two replicates were prepared, giving 12 reference samples all in all. Sample aliquots, 10 g (± 0.05 g), were placed in 50-mL test tubes. Milk of label A was spiked with 19 pesticide model contaminants at four different concentrations (400, 100, 25, and 5 μg/kg) in duplicates, giving eight spiked samples all in all. To obtain the spiked samples either 160, 40, 10, or 2 μL standard solution (25 μg/mL in methanol) was added to 10 g of sample and the sample was vigorously agitated. One milliliter water (purified with a Millipore purification system: MilliQ-Integral) and 6 mL acetonitrile with 1% (*v*/*v*) formic acid were added to 1 mL of sample. The extraction conditions were thereby similar to what has earlier been proven to be efficient for similar multiresidue analysis [11, 12]. The sample was mixed and centrifuged (3000×*g*, 10 °C, 10 min). Supernatants were filtered with Mini-UniPrep™ vials and stored at + 5 °C until analysis by UHPLC-TOF.

**Table 1:** Milk samples used in the study

Label	Sample type	Fat content (%)
A, Arla	Used for spiking	1.5
B, Garant	Reference	0.5, 1.5, 3.0
C, Ekologisk	Reference	0.5, 1.5, 3.0

### Instrumentation

The analysis was performed with an Ultimate 3000 RS (Dionex, Sunnyvale, USA) UHPLC system coupled together with a maXis LC-TOF (Bruker Daltonics, Bremen, Germany). The method was developed by Owens et al. [13] and shown to be a sufficient way to perform target or suspect screening for contaminants, and is now implemented in ToxScreener™ supplied by Bruker. The column for the UHPLC system was an Acclaim RSLC 120 (C18, 2 μm, 2.1 × 100 mm from Dionex) tempered at 30 °C. A gradient for the mobile phase from 11 to 100% methanol in water, with 5 mM ammonium formate and 0.02% formic acid, was used together with a flow gradient from 200 to 450 μL/min (see Table 2 for details). Bottle A contained 0.315 g ammonium formate (> 99%, Fluka), 900 mL water from a Millipore purification system (MilliQ-Integral, EMD Millipore, MA, USA), 100 mL methanol (LiChrosolve, Merck, Darmstadt, Germany), and 200 μL formic acid (pa, Merck). Bottle B contained 0.315 g ammonium formate, 1000 mL methanol, and 200 μL formic acid. As already mentioned, each sample was extracted in duplicates. Each such duplicate was injected twice, thus leading to 40 injections all in all for the study. The injection volume was 4 μL. The resolution for the TOF-MS was set to 20,000 (full width at half maximum at *m/z* = 922). The data was collected from *m/z* 50 to *m/z* 800 in positive mode at 2 Hz. Mass calibration was performed in three steps according to the manufacturer’s instructions. First, a rough calibration with Na(NaCOOH)_*n*_ clusters was performed. With the same calibrant, a fine calibration at each injection was done. Finally, methyl stearate was used as lock mass. The TOF-MS settings were as following: end plate offset − 500 V, capillary − 4500 V, nebulizer (N_2_) 2.4 bar, dry gas (N_2_) 8.0 L/min, dry temperature 190 °C, funnel radiofrequency (RF) 400 V peak-to-peak (Vpp), multipole RF 200 Vpp, ion cooler RF 35 Vpp, transfer time 37 μs, and prepulse storage time 5.0 μs. TracMass 2 was used for data processing, which consisted of two major steps: feature detection (sometimes referred to as “peak picking”) and alignment. The procedure was described in detail in 2014 by Tengstrand et al. [14]. Optimized parameters for TracMass 2 were for “tracking:” minLength 9, minIntensity 200, mzTolerance 0.01, mzAnchor 400, and mzTransformation “sqrt,” and those for “detection;” zafSigma 2, zaf2Sigma 2, gaussSigma 0.375, nSignaltoNoise 15, and stdFiltWidth 16. The part of the software that performs the generalized fuzzy Hough transform, developed to resolve ambiguities in the alignment, was not used since it was regarded unnecessarily due to the stability of retention times in the obtained raw data. The term “feature” is in the present paper defined as a monoisotopic peak from any ion. The output from TracMass 2 is a list of aligned features where not even isotopes from a single ion are clustered. Such lists were exported to Excel, where further sorting of the features was performed according to the criteria described below.

**Table 2:** Gradient for the UHPLC system

Time (min)	Flow (mL/min)	%B
0.0	0.20	1.0
0.1		1.0
1.0	0.20	
3.0		39.0
14.0	0.40	99.9
16.0	0.48	99.9
16.1		1.0
19.0	0.48	
19.1	0.20	

## Results and discussion

### Study design

The aim of the present study was to optimize and investigate an LC-TOF-based system for non-target analysis of unknown or unexpected contaminants in food. The approach was to spike milk samples with compounds normally not present in milk and examine how efficient the system could indicate unique compounds in the spiked samples compared to normal samples (hereafter called reference samples). In a real case, the label of a suspected sample might not be known, or reference samples of the same label as the suspected sample might not be available. In order to mimic such cases, no reference samples based on label A were used in the study. The goal was to detect as many as possible of the added compounds, or—more exactly—to detect as many features (protonated ions, adducts, fragments, or any isotope of these) as possible originating from the added compounds, here referred to as “true positives,” and at the same time avoid as many “false positive” signals as possible, i.e., any signal that did not origin from any of the added compounds. No databases or target lists of compounds were used. Positive detection solely relied on detection of features/signals that were present in (all injections of) the spiked sample but not present in (any injection of) the reference samples. Methods for identification or further analysis of the compounds were outside the scope of the study.

It was earlier noticed in *suspect* screening of contaminants in food at our laboratory—using the same methods (extraction and chromatography) and equipment—that the search for listed compounds comprising no more elements than C, H, N, and O rendered far more false positive hits than if also other elements (e.g., S, P, and Cl) were included in the searched formula. In fact, the intensity threshold in the data processing software could generally be set at least one order of magnitude lower for the latter formulas, still not receiving a higher false positive rate than for the “CHNO-formulas.” The observed effect might be related to differences in isotopic pattern or mass defects compared to the majority of naturally occurring compounds. Consequently, it was decided to include compounds with a variety of elemental composition for spiking of the milk samples in the present study to reveal any similar effect for the *non-target* approach (see Table 3).

**Table 3:** Elemental composition of the pesticides used for spiking of milk

Name	Formula	Number of nitrogen atoms	Elements other than C, H, N, and O
Acephate	C4H10NO3PS	1	P, S
Omethoate	C5H12NO4PS	1	P, S
Dimethoate	C5H12NO3PS2	1	P, S2
Paraoxonmethyl	C8H10NO6P	1	P
Dichlorvos	C4H7Cl2O4P		Cl2, P
Fenthion-sulfon	C10H15O5PS2		P, S2
Atrazine	C8H14ClN5	5	Cl
Metalaxyl	C15H21NO4	1	
Methidathion	C6H11N2O4PS3	2	P, S3
Triadimefon	C14H16ClN3O2	3	Cl
Prometryn	C10H19N5S	5	S
Fenarimol	C17H12Cl2N2O	2	Cl2
Tebuconazole	C16H22ClN3O	3	Cl
Chlorfenvinphos	C12H14Cl3O4P		Cl3, P
Fenthion	C10H15O3PS2		P, S2
Diazinon	C12H21N2O3PS	2	P, S
Propiconazole	C15H17Cl2N3O2	3	Cl2
Prochloraz	C15H16Cl3N3O2	3	Cl3
Ethion	C9H22O4P2S4		P2, S4

The “false positive” signals mentioned above can be classified as one out of two main categories. Either they are (a) system-related, i.e., features that were not unique for the spiked sample but falsely indicated so due to, e.g., sudden baseline shift, severe drift in retention time, or mass calibration, or caused by the software due to non-optimal parameter settings for the algorithms, etc. Or they are (b) reference-related, i.e., due to naturally occurring compounds that were truly unique for the suspected sample when compared to the reference samples. In that case, the reference samples were too few, or not representative enough.

### Optimization

While extraction and the LC-TOF method already were regarded as optimized, as explained above, the data processing step was identified as the crucial part needing optimization. There are various software available for the job, e.g., XCMS [15], MetAlign [16], MZmine 2 [17], or other as reviewed by Katajamaa and Orešič [18]. TracMass 2 was chosen due to a combination of its well-developed graphical interface and a proven efficiency in metabolomics [14]. A data set with spiked and non-spiked juice samples, analyzed using the same analytical method [10] as for milk in the present study, was used for optimization of the parameter settings. The obtained visualized feedback by TracMass 2 facilitated the initial manual optimization. Experimental design was used to find the optimal combination of parameter settings for three crucial parameters: zero area filter, signal to noise, and standard filter width. The optimization target was to have as many true positives as possible and as few false positives as possible. The result for the 26 added compounds was an increase of the total number of detected features from 141 to 191 compared to the previous study [10] (see Table 4). The optimized parameters (see instrumental) were thereafter applied when analyzing the milk samples.

**Table 4:** Number of features found before and after optimisation using the dataset for orange juice (see text)

Compound	Number of detected features in the old study [10]	Number of detected features after optimisation
Aflatoxin G2	3	3
Aflatoxin G1	2	3
Aflatoxin B2	3	3
Aflatoxin B1	2	2
Diacetoxyscirpenol	3	4
T-2 Mycotoxin	1	1
Sterigmatocystin	3	3
Sulfadoxin	14	22
Acephate	5	5
Omethoate	6	8
Dimethoate	9	10
Paraoxonmethyl	4	4
Dichlorvos	4	4
Fenthion-sulfone	7	9
Atrazine	5	5
Metalaxyl	8	9
Methidathion	4	8
Triadimefon	3	11
Fenarimol	2	5
Tebuconazole	2	9
Chlorfenvinfos	14	16
Fenthion	5	6
Diazinon	3	4
Propiconazol	5	6
Prochloraz	11	16
Ethion	13	15
Sum	141	191

### Application to milk samples/detection capability in milk

Preliminary results showed that a reasonable number of reference samples (i.e., unspiked milk samples) were 6–9, extracted in duplicates and each extract analyzed twice. This number compensated for variations in milk composition, extraction, chromatography, detection, and data processing, and gave a manageable number of analyses to handle. Three labels of milk, with three different fat contents, were therefore included in the study (Table 1).

The 19 pesticides chosen as model compounds to mimic unknown contamination were identical to the compounds in the previous study on orange juice [10], thus facilitating comparison of the results between the studies. They were all “LC-pesticides,” meaning that they were already classified by the laboratory to at least be more suitable for LC/MS than for GC/MS. In order to be able to make general conclusions of the methods applicability in any food crisis or for any unexpected contamination, not focusing on a specific scenario, the compounds were chosen to be as diverse as possible in terms of retention time and molecular weight (see Table 5 for both parameters) and elemental composition (see Table 3).

**Table 5:** Detailed results for the spiked components. All detected unique features, including adducts, isotopes and fragments, were counted for each spiked substance. For the *number of detected substances*, each substance was considered where at least one feature was detected

Compound	Retention time (min)	[M+H^+^]^+^ (*m/z*)	Number of detected true positive features per compound, at the denoted spiking level			
			5 μg/kg	25 μg/kg	100 μg/kg	400 μg/kg
Acephate	2.99	184.0190	0	0	1	1
Omethoate	3.24	214.0303	0	1	1	2
Dimethoate	5.09	230.0069	0	2	4	11
Paraoxonmethyl	6.22	248.0323	1	1	2	4
Dichlorvos	6.81	220.9536	0	1	2	5
Fenthion-sulfone	7.41	311.0170	0	1	2	6
Atrazine	7.98	216.1015	1	2	6	8
Metalaxyl	8.06	280.1586	1	4	6	13
Methidathion	8.53	302.9687	0	2	2	6
Triadimefon	9.53	294.1004	0	1	4	8
Prometryn	9.78	335.0350	1	3	5	6
Fenarimol	9.97	331.0395	0	0	2	4
Tebuconazole	10.73	331.1370	0	4	9	15
Chlorfenvinfos	10.80	358.9770	0	3	4	8
Fenthion	10.84	279.0282	1	1	3	3
Diazinon	10.88	305.1092	1	2	3	9
Propiconazol	11.07	342.0771	0	2	5	12
Prochloraz	11.12	376.0379	0	2	5	12
Ethion	12.28	384.9952	0	2	6	15
Total number of detected true positive features			6	34	72	148
Number of detected substances (maximum 19)			6	17	19	19

The spiking levels of the pesticides ranged from 400 μg/kg—the lowest spiking level (of mycotoxins) in the previous study—down to 5 μg/kg in order to examine the detection capabilities of the presented method. Data evaluation was performed separately for each spiking level in order to make sure that the feature detection and alignment algorithms did not benefit from a higher spiking level than the examined.

**Fig. 1:**
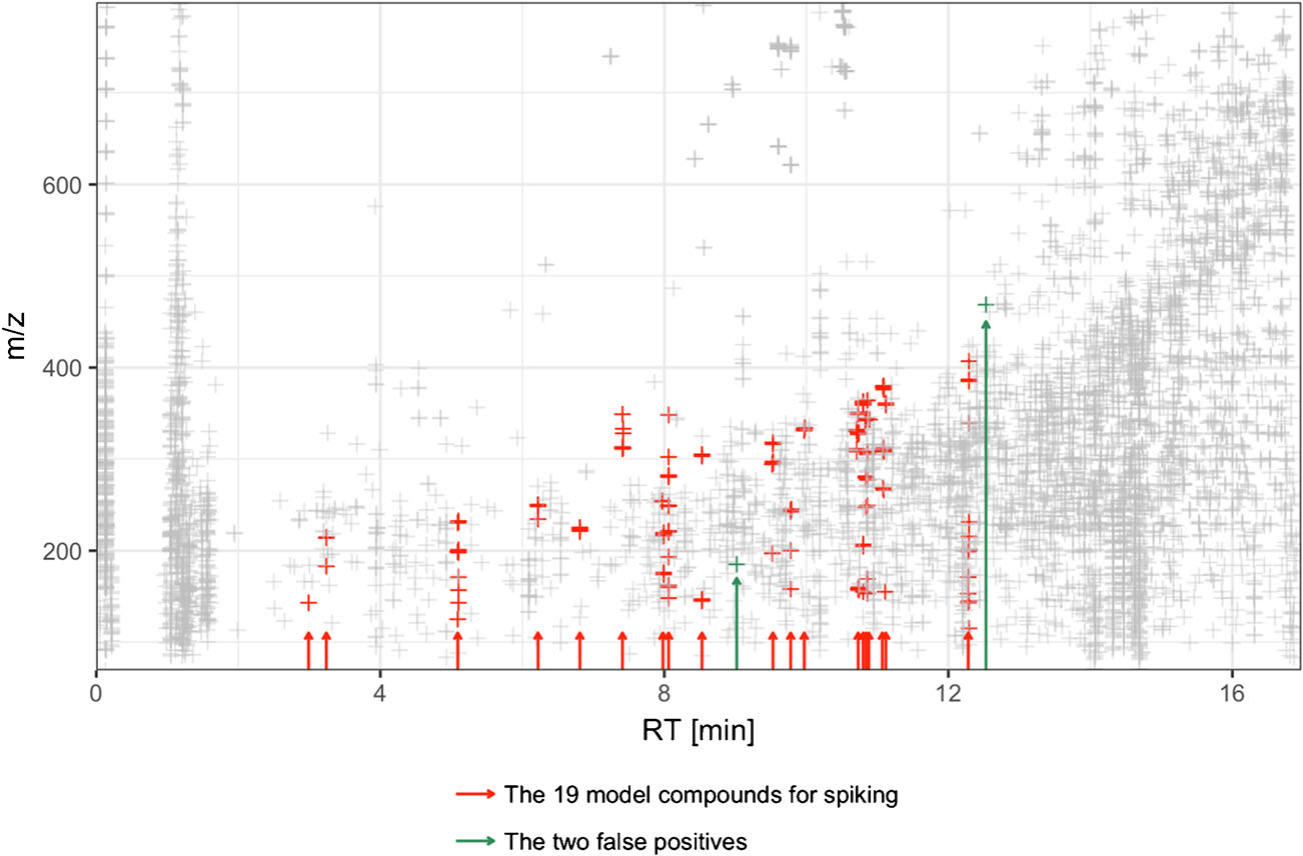
Diagram showing all detected molecular features from an analysis of a milk sample spiked at 400 μg/kg with 19 model compounds. All gray crosses are either matrix features due to naturally occurring compounds in the milk sample, or they are features appearing in the background originating from the equipment or the used solvents. They were found also in at least one of the reference samples (i.e., the unspiked milk samples) and did therefore not qualify as positive hits. All red and green crosses are features that were detected in the spiked sample only, and consequently, they qualified as positive hits. The red crosses originated from the 19 added model compounds. However, the two green features could not be assigned to any of the 19 compounds; they were probably due to naturally occurring compounds occurring in the spiked sample only and are therefore regarded as false positive hits. The example illustrates the complexity in finding unexpected unique compounds and why it is necessary to use computational automatic feature detection as described in the present work

In general, approximately 5500 features were detected in a milk sample by the system. The majority of these features were either matrix features, i.e., they aroused from naturally occurring compounds in the milk samples, or they were background features originating from the solvents or the equipment used. The criteria for any of the features to be regarded as a positive hit was that it should be detected in all four injections of any of the spiked samples (2 extractions × 2 injections) and not in any of the 24 injections of the reference samples (2 varieties × 3 fat concentrations × 2 extractions × 2 injections). The criteria can obviously be set in other ways; however, the idea was to have strict criteria to avoid false positives and to be able to automate the entire process up to this point without doing any manual inspection. Hence, the results before doing any complementary manual evaluation are presented in Tables 5 and 6. For a typical result from an injection of a spiked sample, see Fig. 1. (Calculated data, chromatograms, spectra, mass accuracy, adducts, etc. are included in the Electronic Supplementary Material (ESM).)

**Table 6:** False positive features (see text)

Retention time (min)	*m/z*	Comment
1.61	142.9925	Peak splitting of acephate^a^
4.36	182.9874	Spike contaminant^b^
8.17	342.9984 and 344.9959	Spike contaminant^b^
8.99	230.1162	Spike contaminant^b^
9.02	185.1535	Label A unique^c^
12.43	241.2156	Traces discarded after manual inspection^d^
12.53	468.3079	Label A unique^c^
15.36	314.3047 and 298.2819	Spike contaminant^b^
15.82	628.5504	Traces discarded after manual inspection^d^

^a^Peak splitting of the most hydrophilic compound in the study. Only noticed at 100 and 400 μg/kg

^b^Spike contaminants originating from the standard solution, only noted at 400 μg/kg

^c^Label A unique features were all at two to three times above the noise level only. Probably naturally occurring compounds that could only be detected in the “Arla 1.5% fat sample”

^d^Traces discarded after manual inspection, since it revealed that the same traces were also present in one or several of the reference samples, but at such low concentration, so the peak detection algorithm had not detected the peak

**Fig. 2:**
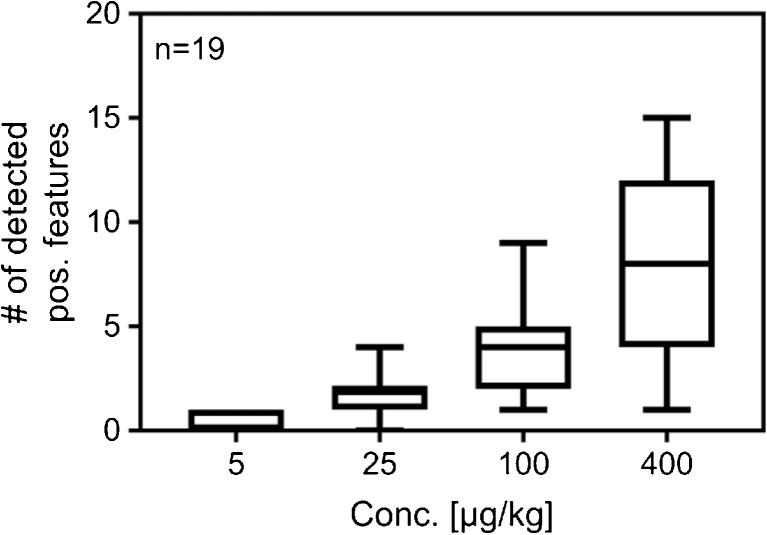
Boxplot showing the number of detected features per added model compound. Mean, standard deviation, and min/max for the 19 compounds are plotted versus the added concentration of the model compounds

Table 5 shows that all 19 model contaminants could be detected in the 400- and 100-μg/kg groups, although the number of detectable features originating from the compounds (“true positives”) was reduced by approximately 50% at 100 μg/kg compared to 400 μg/kg (see also Fig. 2). Also in the 25-μg/kg group, a majority of the spiked compounds were detected (17 out of 19) and most of them by more than one feature. However, in the 5-μg/kg group, only 6 out of 19 added substances were detected by the search algorithm. In other words, the biggest loss in detectable model contaminants occurred between 5 and 25 μg/kg. This would be in the same order of magnitude as the detection limits commonly required in the EU for routine target screening of food contaminants [19, 20]. Typical intensities for features detected at 25 μg/kg but not at 5 μg/kg were, e.g., 755 ± 163, 865 ± 169, 454 ± 21, and 365 ± 26 (mean ± sd) counts per second (cps) for triadimefon, fentionsulfon, dichlorvos, and omethoate, respectively, at 25 μg/kg. These observed intensities were close to the intensity cutoff (150 cps) which was slightly above the noise level. The expected intensity—when taking the standard deviation into account—of the mentioned compounds at 5 μg/kg did thereby not exceed the cutoff level and/or noise level significantly. This indicates that the algorithms utilized the data effectively, and that further optimization of the algorithms or the settings in the software would not improve the results significantly. The recommended intensity threshold is 200 cps when using the same instruments and methods for *targeted* analysis. The results of the present study showed also in this way that non-targeted analysis reached similar low detection levels as targeted analysis when using the same instruments and methods.

The number of nitrogen atoms in the model compounds varied from zero to five (see Table 3). The two most nitrogen-rich compounds were among the few compounds that were detected even at the lowest spiking level, 5 μg/kg (see Table 5). This indicates that nitrogen-rich compounds would at least work as well as other compounds when using the method. Compounds with more than one Cl were not detected at the lowest spiking concentration, but were detected with as many, or in average even slightly more, features than other compounds at the highest spiking level. These results might both be explained by the fact that the obtained signals are split on several Cl isotopes. Compounds with a molecular weight > 300 Da were detected with in average 50% more features at 400 μg/kg than compounds < 300 Da. No other clear correlation was observed between the number of found positive features and retention time, molecular weight, or elemental composition.

Table 6 shows the “false positive” hits, i.e., any detected feature that did not origin from the added compounds. In total, 11 features were false hits and were inspected manually. One of these features was due to peak splitting of an added pesticide and six were found to be due to contaminants originating from the standard solutions used for spiking. Since these features were not from the milk, these “false positives” can be seen as unexpected contaminants. Thus, only four false positive features remained and had been present in a real case. Out of these four, all at a low intensity, two were quickly discarded (intensity 600–3100 cps) since they also occurred in one or several of the references, but too low to have been detected by the peak detection algorithm (i.e., system-related false positives, see above). The other two (intensity 150–400 cps) could not be discarded in the same way and were either unique for the A sample, or the intensity too low in the references to even be seen at manual inspection (i.e., reference-related false positives, see above and see also Fig. 1).

As a general conclusion, a practical detection limit (LOD), or lower limit of working-range, was established at around 25 μg/kg since most compounds (17 out of 19) were detected at this concentration without receiving more than two system-related and two reference-related false positives.

In order to examine if a smaller number of reference samples—or fewer analyzes of them—still would give acceptable results parts of the data were excluded prior to data processing by TracMass 2. Either all duplicate injections were excluded, or all duplicate extractions or all reference samples of label A or label B. The results showed that a similar amount of true positive features were detected. However, regardless of which part of the data that was excluded, the amount of false positive features was two to three times higher.

A possible way of improving the detection capability of the method, i.e., to achieve an even lower limit of working-range, would be to decrease the dilution during extraction, or to increase the injection volume. However, since this might influence the stability of the chromatography or the ionization efficiency, and thereby hamper proper alignment or decrease the repeatability in the intensity evaluation, any improvement of the final results remains to be proven.

Limit of quantification (LOQ) was not evaluated for the method since it is regarded as a qualitative method if used as described.

### Discussion

The method was initially developed to be used for investigations in a crisis situation, e.g., a severe intoxication. For acute toxic effects after oral administration of many well-known toxic compounds, relatively high concentrations are needed. For example, saxitoxins—a group of marine biotoxins and among the most toxic compounds known—have an estimated oral LOAEL (lowest observed adverse effect level) of 1.5 μg saxitoxin equivalents/kg body weight (BW) for humans and a lethal dose at least one order of magnitude higher [21]. For 1 kg of a contaminated food item and a BW of 70 kg, 10–50 μg/kg BW would correspond to 700–3500 μg/kg.

The achieved low detection capability of 25 μg/kg is far below the level above but might be needed for investigations of suspected contaminations since (a) sampling might not have covered the most contaminated part of the food, (b) the compound might be unstable, and (c) the investigation was initiated due to other reasons than symptoms (threat, strange appearance, accident, etc.). Moreover, as discussed in the Introduction, sensitive methods would enable monitoring for new or unexpected contaminants on a regular basis, as suggested by Bader et al. for water [22, 23]. Although the melamine scandal involved positive samples of liquid milk and yogurt in a very high concentration interval of 2.5 to 10 mg/kg [3], reports of “emerging risks” more often include considerably lower concentrations and hence, sensitive methods would be beneficial, as in the mentioned Swedish PFAS scandal where only up to 10 μg/L in outgoing drinking water was reported [4]. The applicability of the method to other matrices than milk will probably be highly dependent on the interference of the food matrix compounds, the critical point being the variation between samples within a food group, and hence whether representative food reference samples are available. The detection capability has therefore to be evaluated for each food matrix separately.

Although a semi-automatized data evaluation method was developed, the suggested screening approach will require more work and resources than regular routine methods and will therefore not be viable for a high number of samples. However, it would be valuable to use the non-targeted method as complementary to the targeted routine screening efforts. Another possibility would be to use the presented non-target screening approach in combination with cheaper broad screening methods that do not even need to be informative in its results. One example would be the “raw milk untargeted adulteration screening” performed by FTIR technology (Fourier transform infrared spectrophotometry) already in use at Chinese dairies [24]. The benchtop technique can classify samples as being normal or abnormal due to unknown contamination with detection limits ≥ 300 mg/L for various organic compounds. The technique, although not very sensitive, could be combined with further analysis of the abnormal samples by the presented non-targeted HRMS methodology.

Recent publications describing the use of HRMS and a metabolomics approach for food analysis often include classifications, either based on geographical origin or food type [25], or in order to reveal possible adulteration [26]. The described tools are commonly based on multivariate statistics. Fewer attempts have been made to use HRMS and foodomics in order to determine the presence of contaminants, as reviewed by Knolhoff and Croley [27]. One such a recent study with promising results [28] has great similarities to the present study. Main differences are that their reference samples were all of the same type and from the same manufacturer as the suspect sample, and that our work involved a broader range of model compounds. Moreover, the present work was based on a simpler workflow, not making use of multivariate statistics, and did not rely on instrument vendor software. The results are though very similar in terms of apparent limit of detection and number of false positives. This should encourage others to further improve the workflows, or develop their own alternatives, in order to meet the demand of non-targeted contaminant detection in foods.

Current state-of-the-art screening methods for contaminants using UHPLC-TOF are capable of detecting a large number of compounds, e.g., 400 in water [29], 600 in food [30], or currently over 1600 for forensic screening [31]. However, all other possible contaminants will still pass unnoticed, which might be any out of millions of organic compounds, since—as an example—there are currently over 50 million compounds registered in ChemSpider [32]. The metabolomics-based non-targeted approach described in the present paper might at least partially fill this gap, especially if applied to both GC-MS and LC-MS, since these techniques together generate signals for most organic compounds. Prioritized signals, i.e., suspect peaks, might thereafter be submitted to compound identification, which was outside the scope of the present study. It should be mentioned that compound identification via molecular formula generation from AM HRMS data can be a tedious work, especially if the elemental composition is identical to many other compounds when searching through databases. A good example is noradrenalin, which at the same time represents a relatively small molecule having physiologic effects on low levels [33]. A formula query using its formula C8H11NO3 rendered 1294 hits in ChemSpider. In such cases, more information would be needed, e.g., retention time, fragmentation information from in-source CID or MS/MS experiments, or even information from other techniques, e.g., NMR. The model compounds used in the study range from 12,696 hits (Metalaxyl, C15H21NO4) to only one hit (Methidathion, C6H11N2O4PS3) in ChemSpider, indicating that compound identification based on molecular formulas can range from virtually impossible to fairly easy. Anyway, even if the chemical identity of a potential contaminant has not yet been fully determined in one or a few samples, the analysis of other samples might aid to track its source or to evaluate its occurrence. Such strategies are already in use in efforts to mitigate environmental pollution. One example is from non-targeted screening of unknowns in the river Rhine. When reference standards were not available for confirmation of identity the point of emission was located by additional upstream sampling and the emitter was asked to provide the chemical [34].

### Conclusions

The optimized non-target method was demonstrated to be suitable for detecting unexpected organic contamination in milk down to 25 μg/kg. The possible application of the method to other food matrices is believed to be highly dependent on the possibility to access relevant reference samples. The method—in many cases more sensitive than needed for investigations of food poisoning due to organic compounds—was suggested to be used as a complement to regular screening surveys to at least get the possibility to reveal completely unexpected food contaminants. If only a fraction of the analyses of contaminants had been carried out in this way the scandals of Chinese milk and Dutch eggs (see Introduction) might have been discovered earlier. The methodology could also possibly be used in combination with cheaper broad screening methods used for detection of abnormal samples, e.g., at diaries, an approach that very well might be applied to other foods or even in other disciplines.

## Electronic supplementary material

ESM 1 (PDF 511 kb)

ESM 2 (XLSX 1473 kb)

ESM 3 (ZIP 2279 kb)

ESM 4 (PPTX 170 kb)
