# Supplementary material for: Non-targeted analysis of unexpected food contaminants using LC-HRMS
Source: Anal Bioanal Chem. 2018 Mar 29;410(22):5593–602. doi: 10.1007/s00216-018-1028-4 (PMC6096699; doi:10.1007/s00216-018-1028-4)
Supplement: Supplementary file 1 — (PDF 511 kb) [file 216_2018_1028_MOESM1_ESM.pdf]

Analytical and Bioanalytical Chemistry

Electronic Supplementary Material

Non-targeted analysis of unexpected food contaminants using LC-HRMS

Marco Kunzelmann, Martin Winter, Magnus Åberg, Karl-Erik Hellenäs, Johan Rosén

Additional files available under 10.1007/s00216-018-1028-4

Content

1. Calculated data from TracMass 2
2. Extracted ion chromatograms
3. Mass spectra
4. Mass accuracy
5. Detected adducts

Comments to Electronic Supplementary Material

1. Calculated data from TracMass 2

The output from the analysis using TracMass 2 is presented in an Excel file (216_2018_1028_MOESM2_ESM.xlsx) and included in the ESM. The table contains, in the first column, the ID number set by TracMass 2 for all detected features in the data set. Thereafter, m/z values and retention times based on the detected features are presented. Finally the calculated area response for each feature is given for each separate analysis. The names of the injection and descriptions of what sample they correspond to are described below.

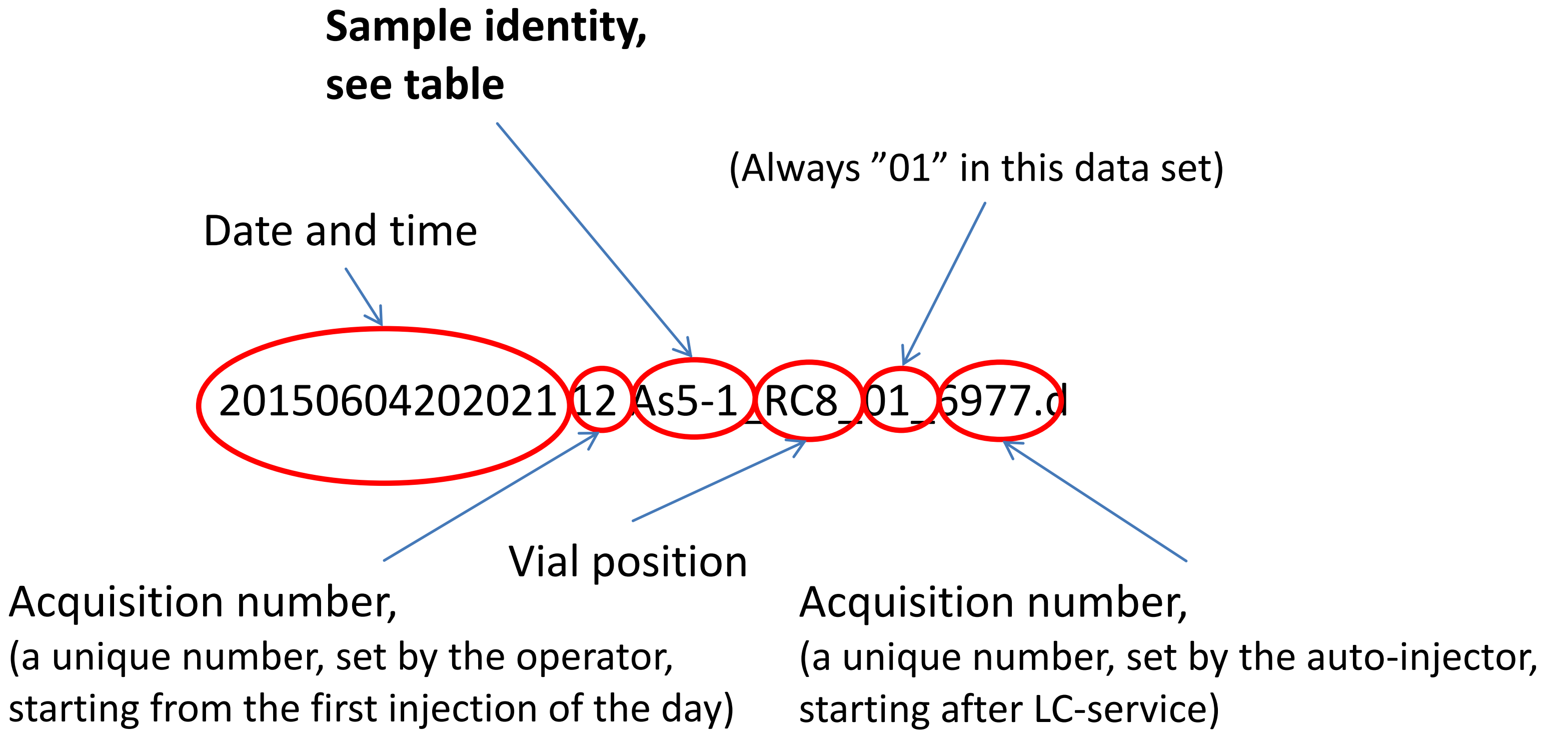

Name	Sample
As5-1	Arla spiked 5ppb 1st extraction
As5-2	…2nd extraction
As25-1	…25 ppb…
…	
E0.5-1	Label “Ekologisk”, 0.5%fat, 1st extraction
E 1.5-1	…1.5%fat…
…	
G0.5-1	Label “Garant”…
…	

2. Extracted ion chromatograms

Extracted ion chromatograms for all spiked components are presented as .png files in the folder EIC_19comp. Retention time is in seconds, and extracted mass range is 6 mDa, time window 30 seconds, missing data points excluded. Separate chromatograms are for the 400 ppb level, while overlay chromatograms are for all four spiking levels.

An extra overlay plot for Dimethoate (Dimethoate overlay 2 minutes.png) with extracted mass range 20 mDa, and time window 2 minutes is presented below. It can be noted that the feature was detected at the three highest spike levels (blue, pink, red), but not at the lowest (green).

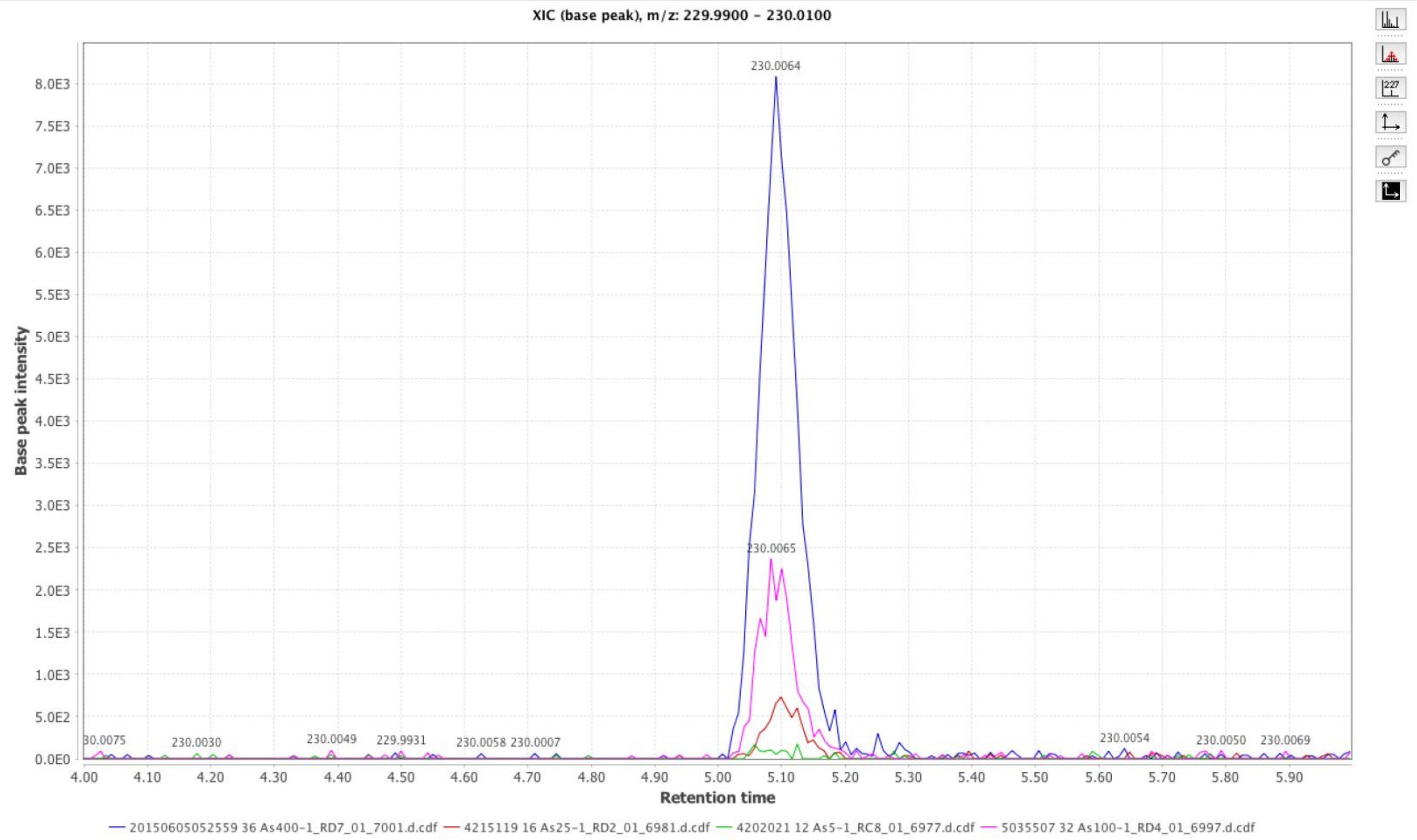

3. Mass spectra

Mass spectra, indicating the detected features with arrows is presented in a ppt-file (216_2018_1028_MOESM4_ESM.pptx). The spectra is a single scan at 400 ppb for the same compound, Dimethoate, as the extra overlay plot above (see "216_2018_1028_MOESM3_ESM.zip"). All features not having an arrow are due to the milk matrix, or are system background components.

4. Mass accuracy

Selected data for the spiked component, inter alia mass accuracy

Compound	Retention time (min)	$[M+H^+]^+$ (*m/z*)	Chemspider ID	Molecular Formula	exact mass (lit.)	mDa	ppm	InChl Keys
Acephate	2,99	142,9924	1905	$C_4H_{10}NO_3PS$	142,9926277	-0,228	-1,592	YASYVMFAVPKPKE-UHFFFAOYSA-N
Omethoate	3,24	214,0295	13574	$C_5H_{12}NO_4PS$	214,030294	-0,794	-3,710	PZXOQEXFMJCDPG-UHFFFAOYSA-N
Dimethoate	5,09	230,0066	2973	$C_5H_{12}NO_3PS_2$	230,00745	-0,850	-3,696	MCWXGJITAZMZEV-UHFFFAOYSA-N
Paraoxonmethyl	6,22	248,0316	13114	$C_8H_{10}NO_6P$	248,032402	-0,802	-3,233	BAFQDKPJKOLXFZ-UHFFFAOYSA-N
Dichlorvos	6,81	220,9528	2931	$C_4H_7Cl_2O_4P$	220,953729	-0,929	-4,205	OEBRKCOSUFCWJD-UHFFFAOYSA-N
Fenthionsulfone	7,41	311,0168	18445	$C_{10}H_{15}O_5PS_2$	311,017682	-0,882	-2,836	ZDHYERRNXRANLI-UHFFFAOYSA-N
Atrazine	7,98	216,1009	2169	$C_8H_{14}ClN_5$	216,101598	-0,698	-3,230	MXWJVTOOROXGIU-UHFFFAOYSA-N
Metalaxyl	8,06	280,1542	38839	$C_{15}H_{21}NO_4$	280,154884	-0,684	-2,442	ZQEIXNIJLIKNTD-

								UHFFFAOYSA-N
Methidathion	8,53	302,9689	13115	$C_6H_{11}N_2O_4PS_3$	302,969687	-0,787	-2,598	MEBQXILRKZHVCX-UHFFFAOYSA-N
Triadimefon	9,53	294,1002	36029	$C_{14}H_{16}ClN_3O_2$	294,10093	-0,730	-2,482	WURBVZBTWMNKQT-UHFFFAOYSA-N
Prometryn	9,78	242,1432	4760	$C_{10}H_{19}N_5S$	242,143942	-0,742	-3,064	AAEVYOVXGOFMJO-UHFFFAOYSA-N
Fenarimol	9,97	331,0396	39394	$C_{17}H_{12}Cl_2N_2O$	331,040494	-0,894	-2,701	NHOWDZOIZKMVAI-UHFFFAOYSA-N
Tebuconazole	10,73	308,152	77680	$C_{16}H_{22}ClN_3O$	308,152965	-0,965	-3,132	PXMNMQRDXWABCY-UHFFFAOYSA-N
Chlorfenvinfos	10,80	358,9762	9703	$C_{12}H_{14}Cl_3O_4P$	358,977357	-1,157	-3,223	FSAVDKDHPDSCTO-XYOKQWHBSA-N
Propiconazol	10,83	342,0768	39402	$C_{15}H_{17}Cl_2N_3O_2$	342,077608	-0,808	-2,362	STJLVHWMYQXCPB-UHFFFAOYSA-N
Fenthion	10,84	279,0271	3229	$C_{10}H_{15}O_3PS_2$	279,027852	-0,752	-2,695	PNVJTZOFSHSLTO-UHFFFAOYSA-N
Diazinon	10,88	305,1077	2909	$C_{12}H_{21}N_2O_3PS$	305,108878	-1,178	-3,861	FHIVAFMUCKRCQO-UHFFFAOYSA-N
Prochloraz	11,12	376,0378	159925	$C_{15}H_{16}Cl_3N_3O_2$	376,038636	-0,836	-2,223	TVLSRXXIMLFWEO-UHFFFAOYSA-N
Ethion	12,28	384,9946	3171	$C_9H_{22}O_4P_2S_4$	384,995449	-0,849	-2,205	RIZMRRKBZQXFOY-UHFFFAOYSA-N

5. Detected adducts

Compound	Adducts, except from H^+
Acephate	-
Omethoate	-
Dimethoate	-
Paraoxon-methyl	-
Dichlorvos	-
Fenthion-sulfon	$NH4^+$, Na^+, K^+
Atrazine	-
Metalaxyl	Na^+
Methidathion	-
Triadimefon	Na^+
Prometryn	-
Fenarimol	-
Tebuconazole	Na^+
Chlorfenvinphos	-
Fenthion	Na^+
Diazinon	-
Propiconazole	-
Prochloraz	-
Ethion	Na^+
