# Supplementary figures and images for: Non-targeted analysis of unexpected food contaminants using LC-HRMS
Source: Anal Bioanal Chem. 2018 Mar 29;410(22):5593–602. doi: 10.1007/s00216-018-1028-4 (PMC6096699; doi:10.1007/s00216-018-1028-4)

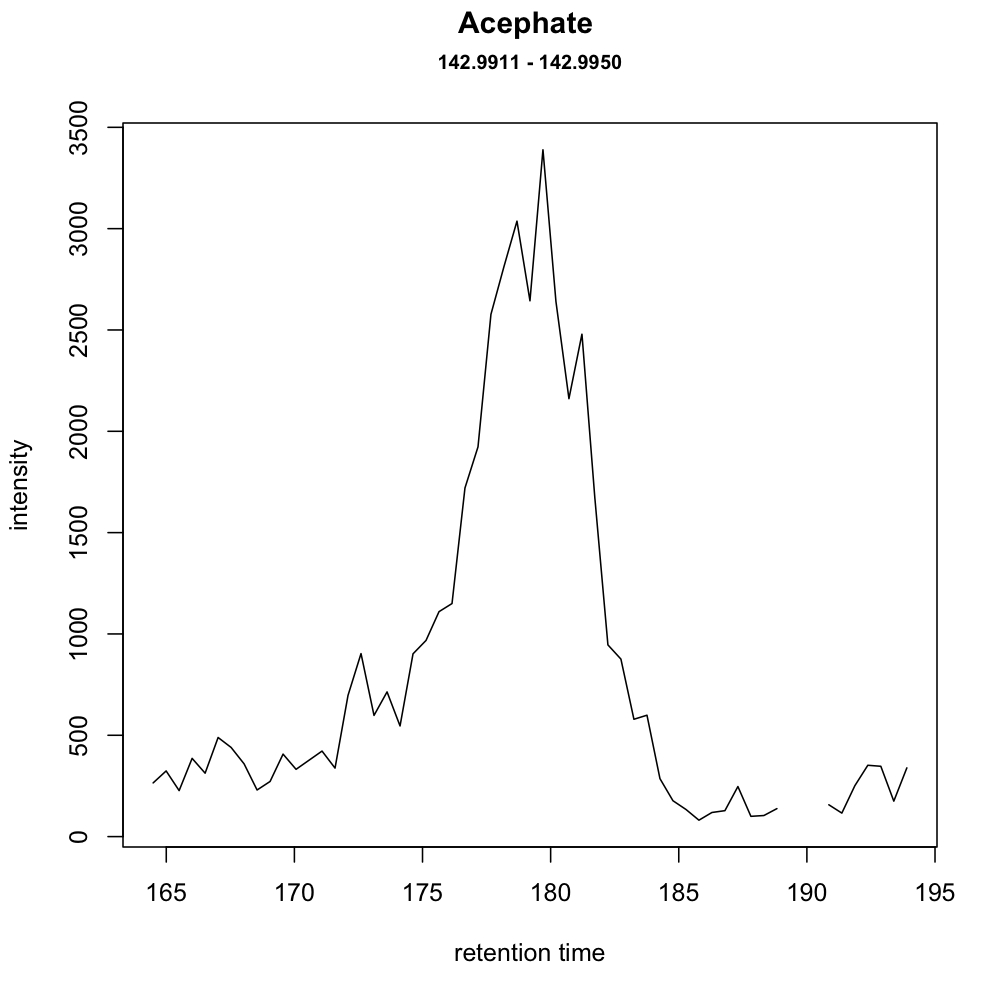

Supplement: Supplementary file 3 — (ZIP 2279 kb) [file 216_2018_1028_MOESM3_ESM.zip › EIC_Acephate.png]

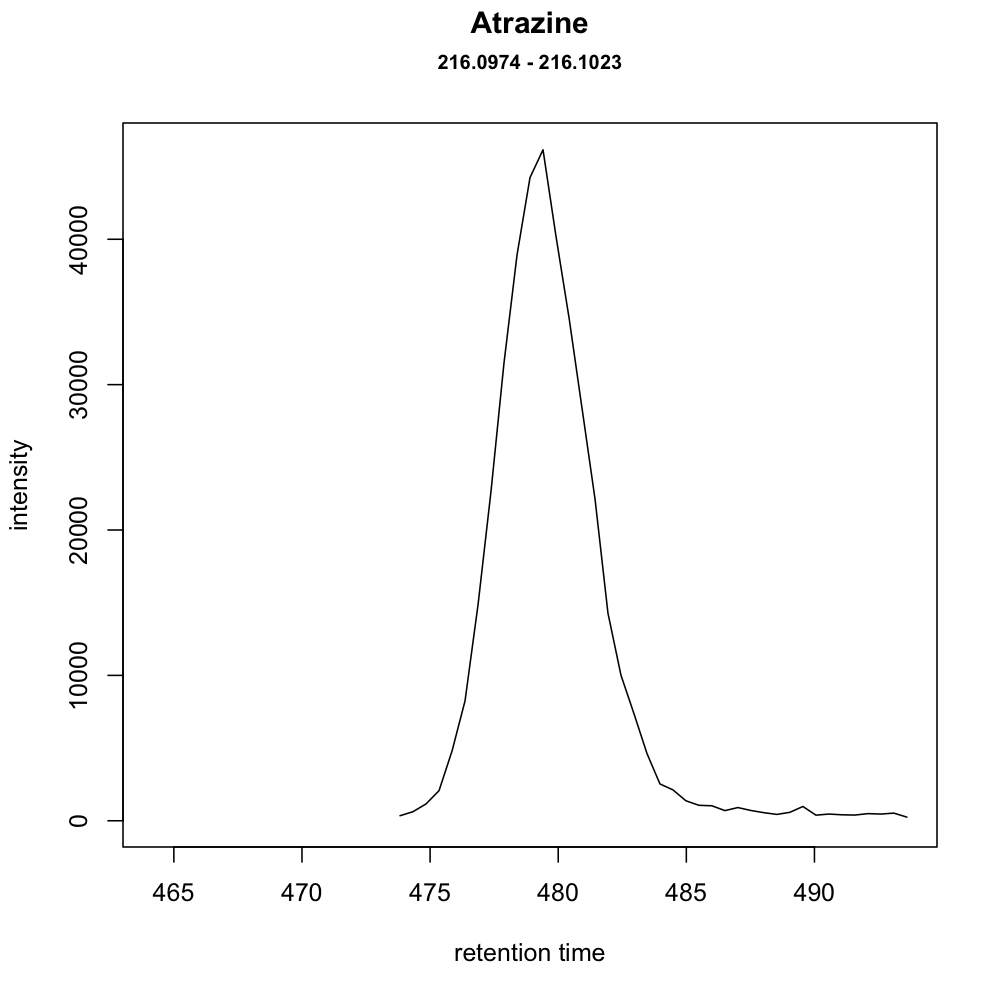

Supplement: Supplementary file 3 — (ZIP 2279 kb) [file 216_2018_1028_MOESM3_ESM.zip › EIC_Atrazine.png]

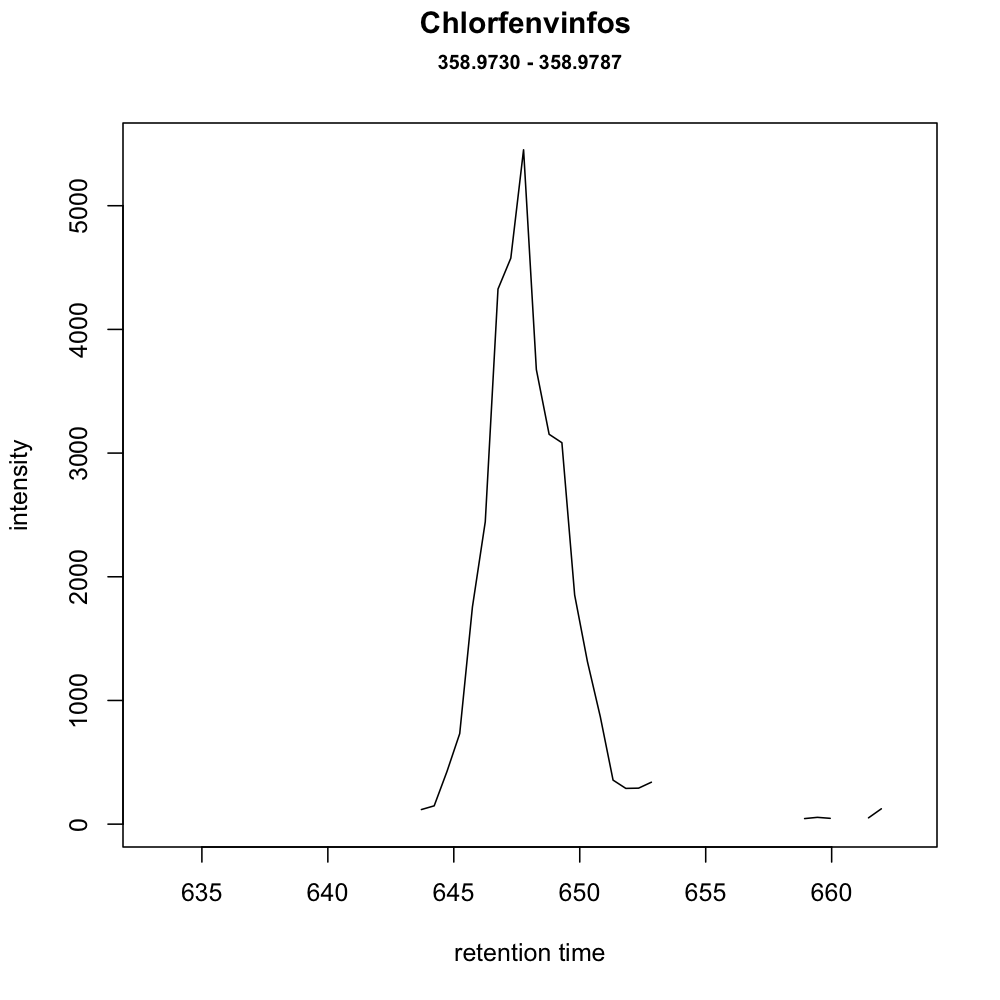

Supplement: Supplementary file 3 — (ZIP 2279 kb) [file 216_2018_1028_MOESM3_ESM.zip › EIC_Chlorfenvinfos .png]

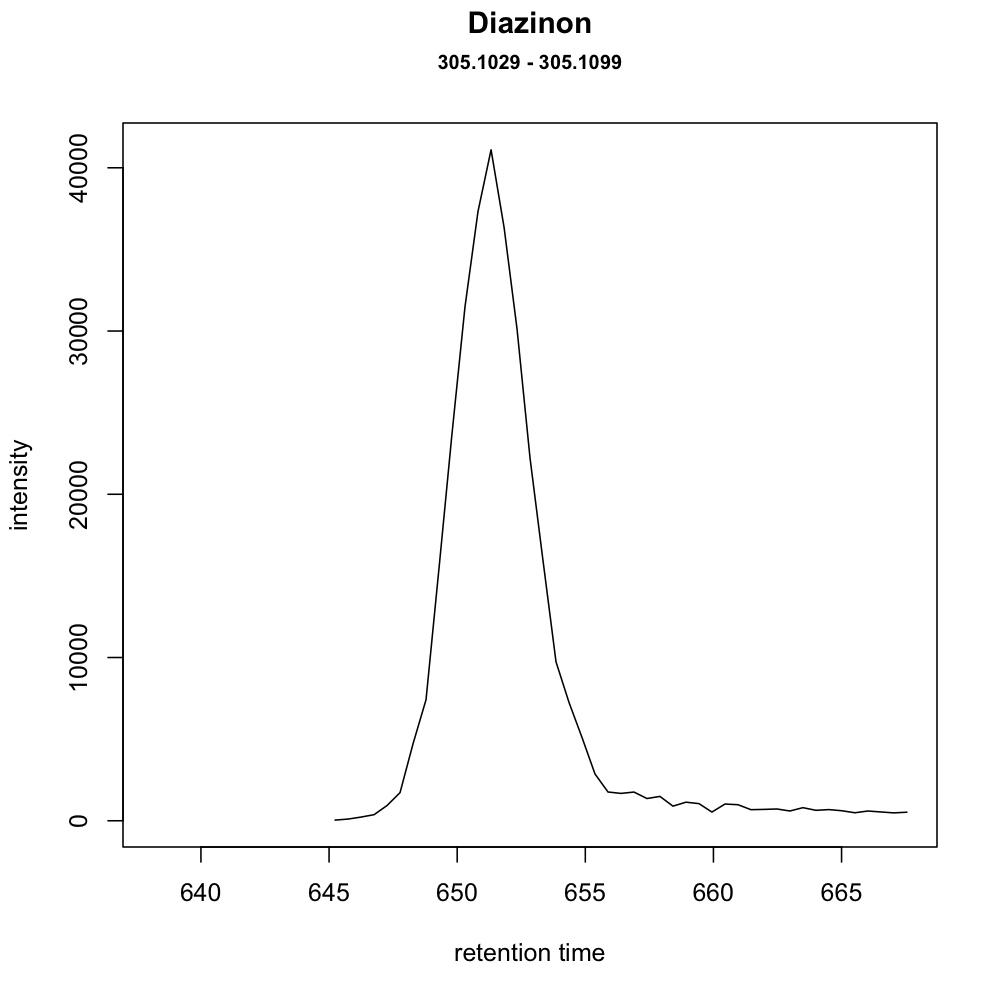

Supplement: Supplementary file 3 — (ZIP 2279 kb) [file 216_2018_1028_MOESM3_ESM.zip › EIC_Diazinon.png]

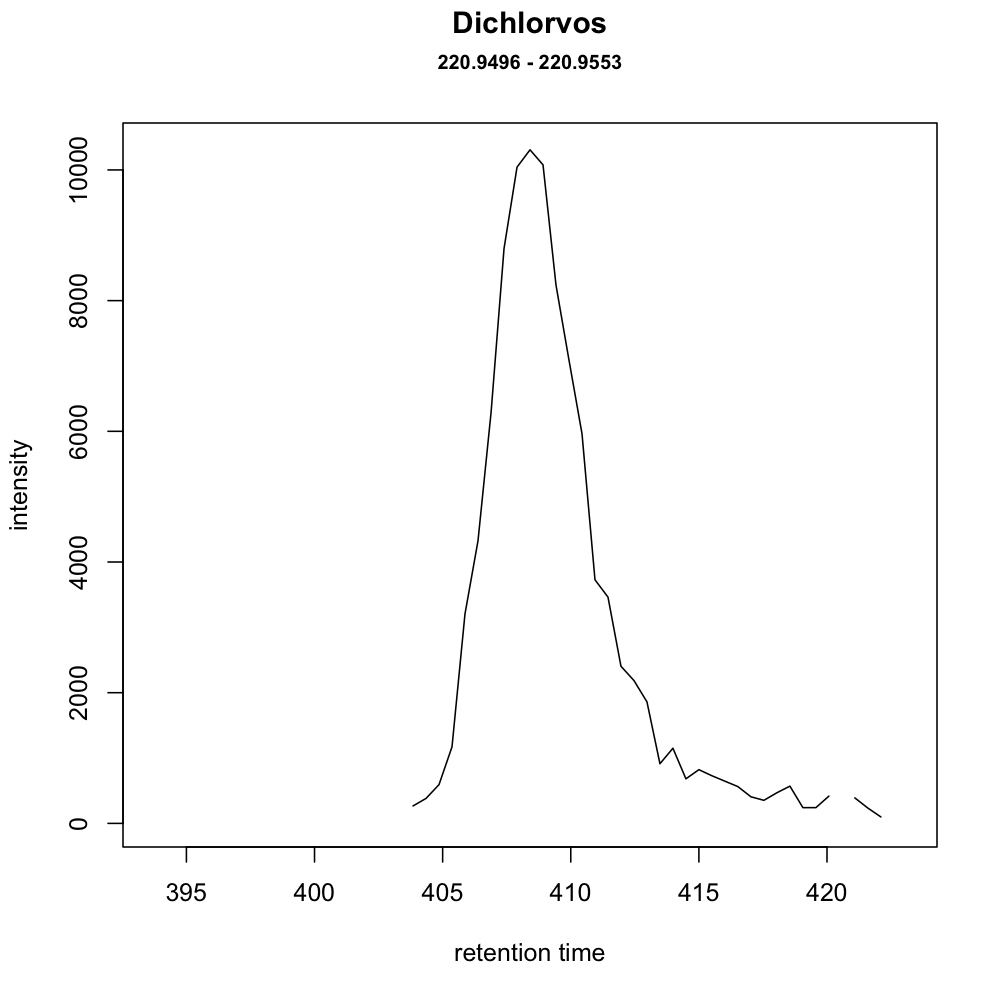

Supplement: Supplementary file 3 — (ZIP 2279 kb) [file 216_2018_1028_MOESM3_ESM.zip › EIC_Dichlorvos.png]

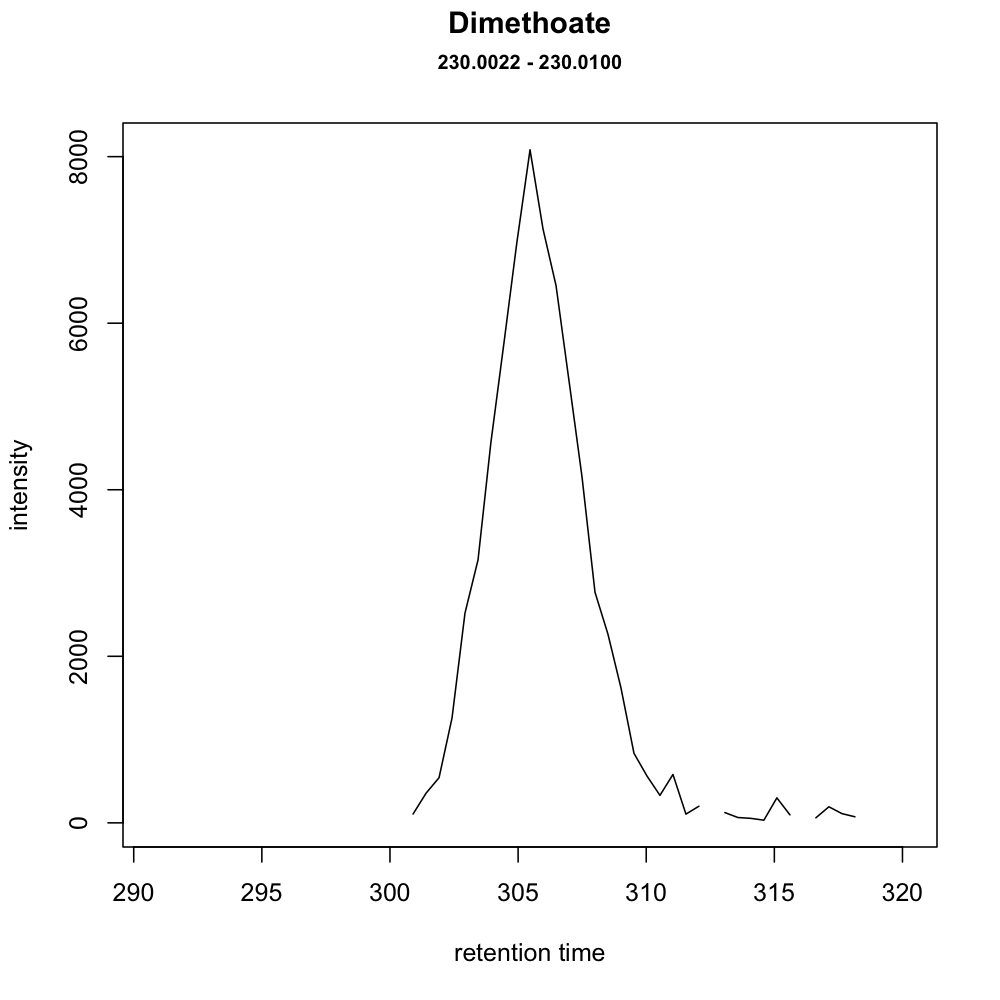

Supplement: Supplementary file 3 — (ZIP 2279 kb) [file 216_2018_1028_MOESM3_ESM.zip › EIC_Dimethoate.png]

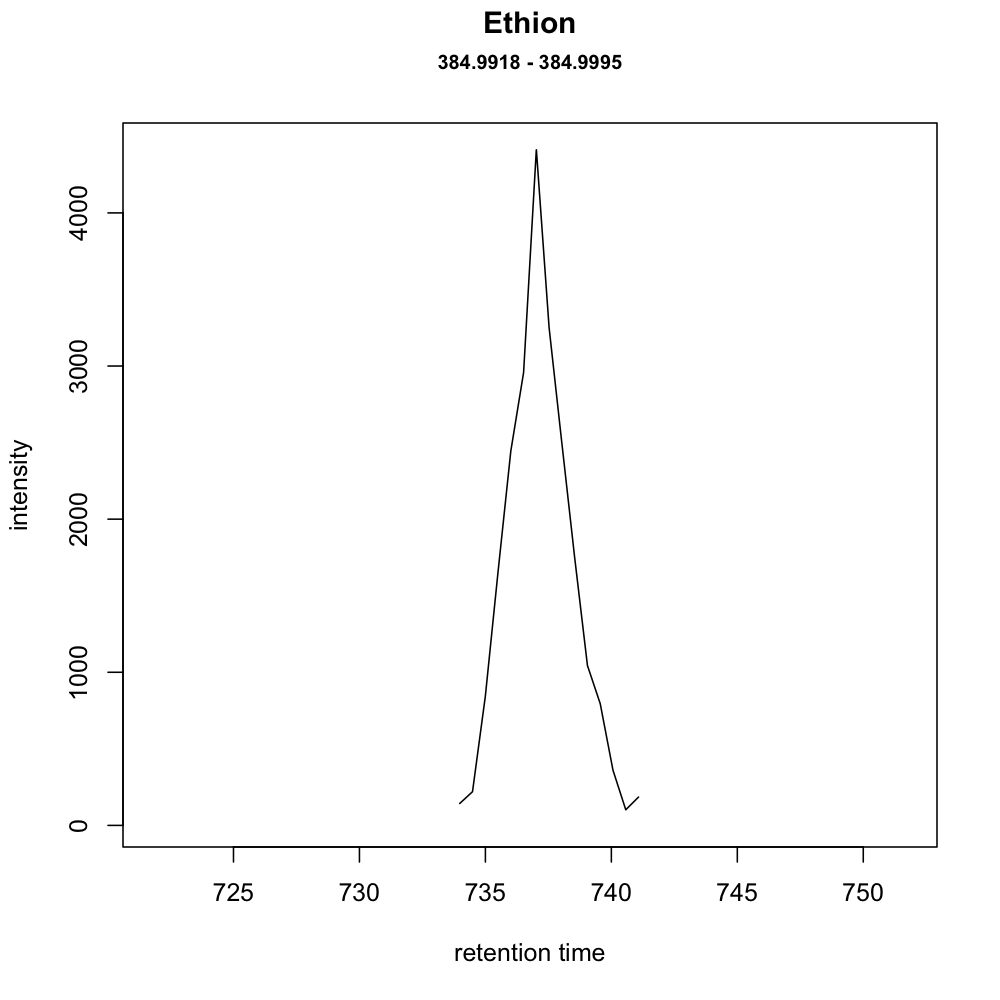

Supplement: Supplementary file 3 — (ZIP 2279 kb) [file 216_2018_1028_MOESM3_ESM.zip › EIC_Ethion.png]

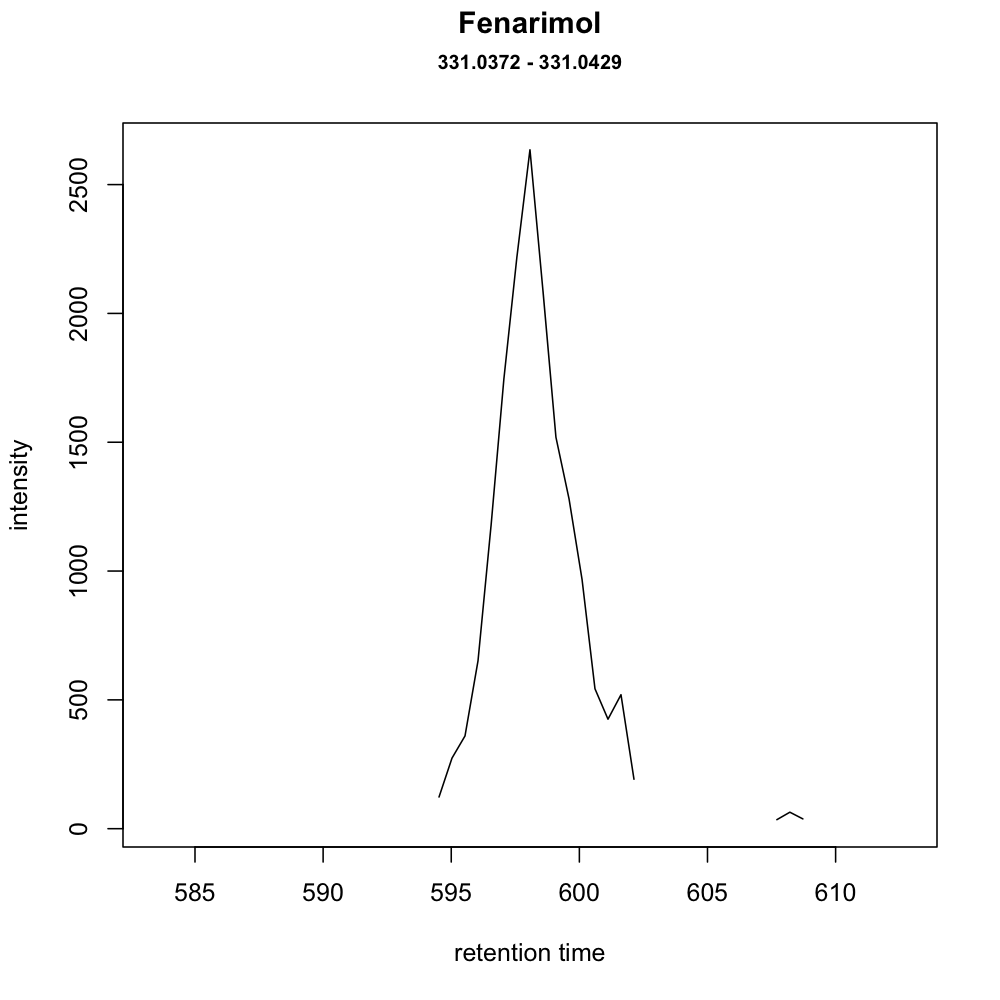

Supplement: Supplementary file 3 — (ZIP 2279 kb) [file 216_2018_1028_MOESM3_ESM.zip › EIC_Fenarimol.png]

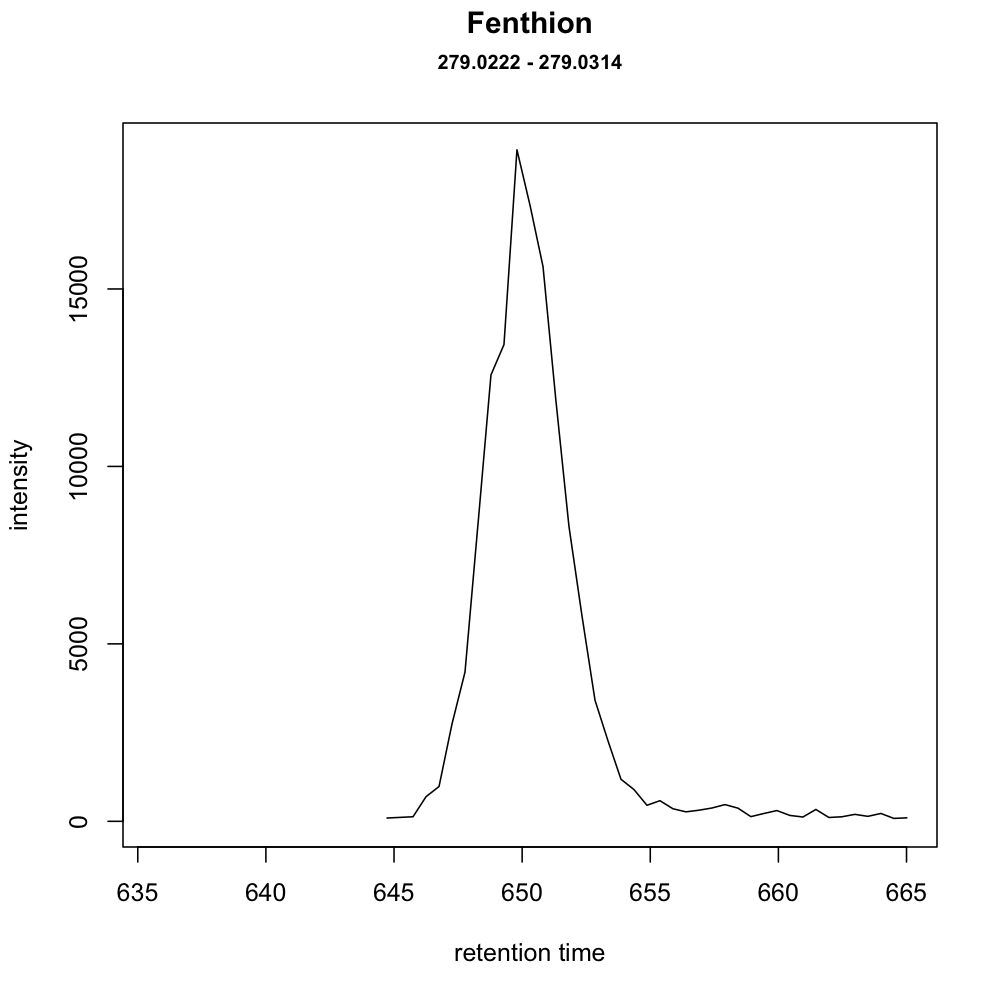

Supplement: Supplementary file 3 — (ZIP 2279 kb) [file 216_2018_1028_MOESM3_ESM.zip › EIC_Fenthion.png]

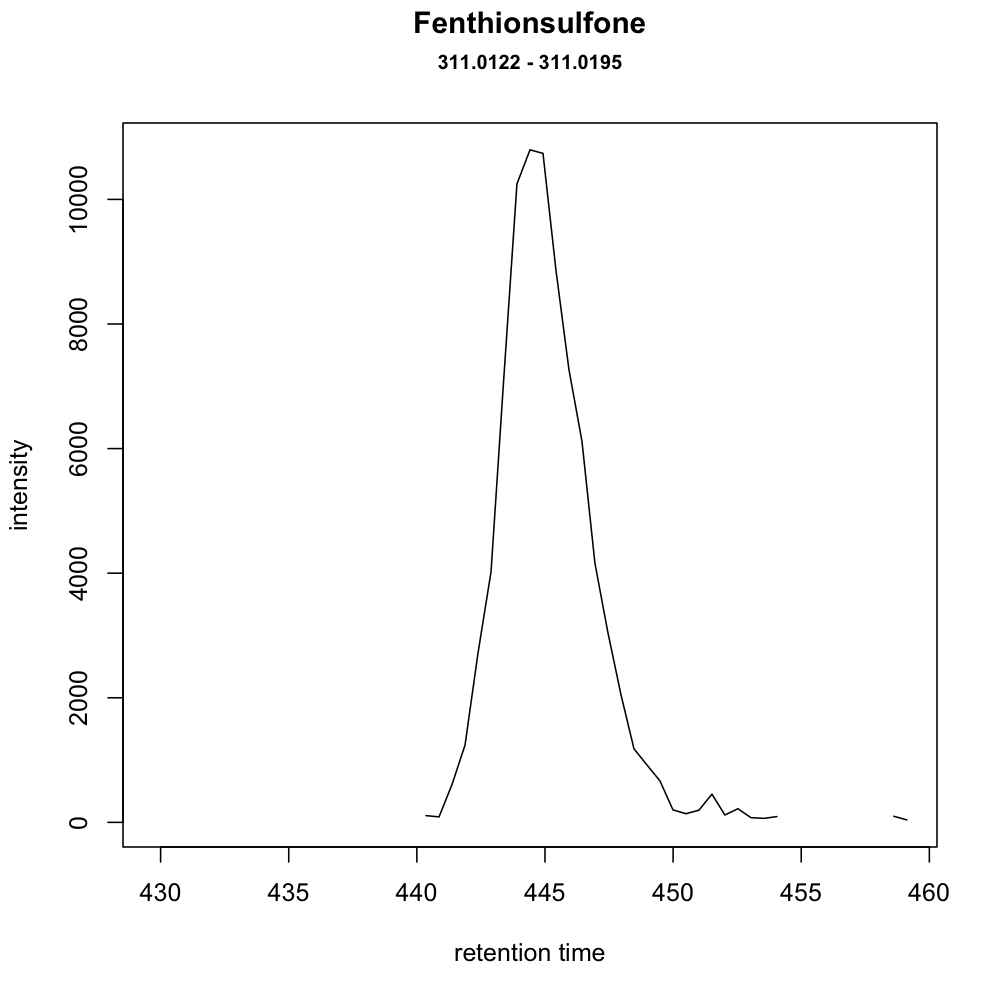

Supplement: Supplementary file 3 — (ZIP 2279 kb) [file 216_2018_1028_MOESM3_ESM.zip › EIC_Fenthionsulfone.png]

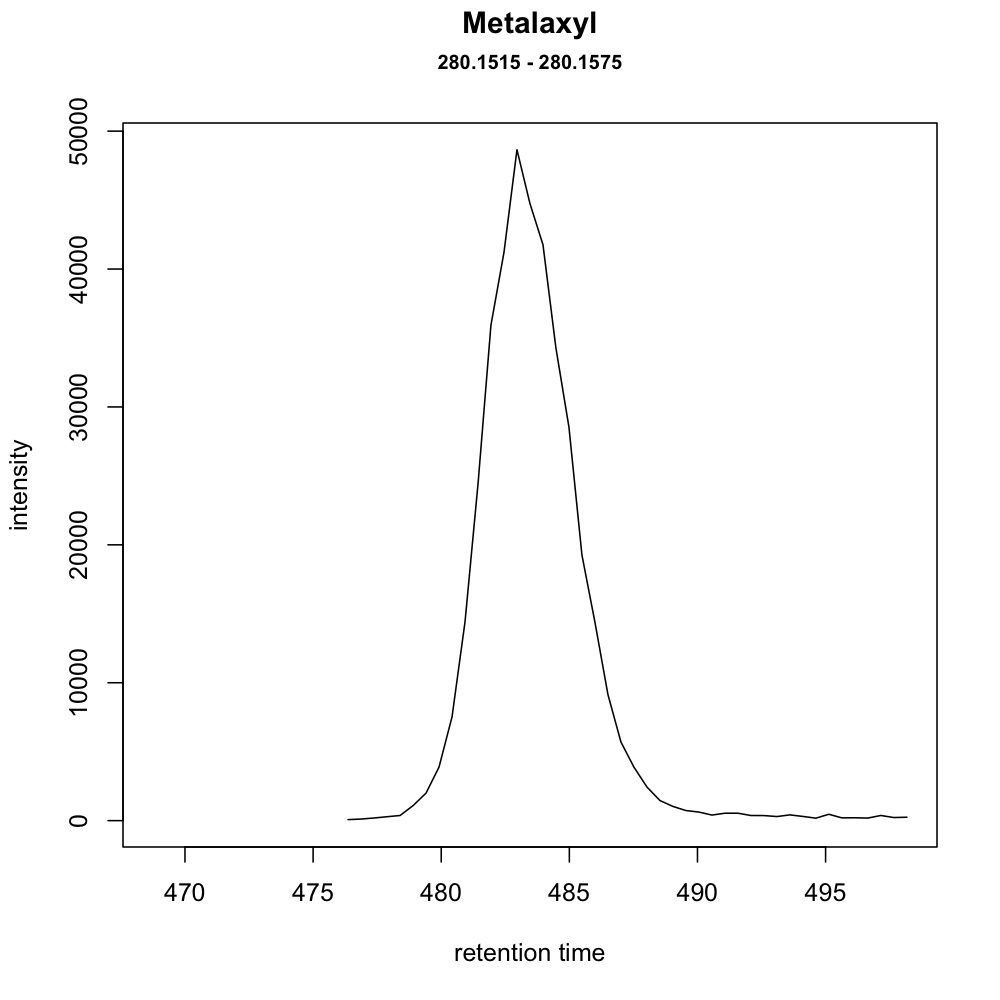

Supplement: Supplementary file 3 — (ZIP 2279 kb) [file 216_2018_1028_MOESM3_ESM.zip › EIC_Metalaxyl.png]

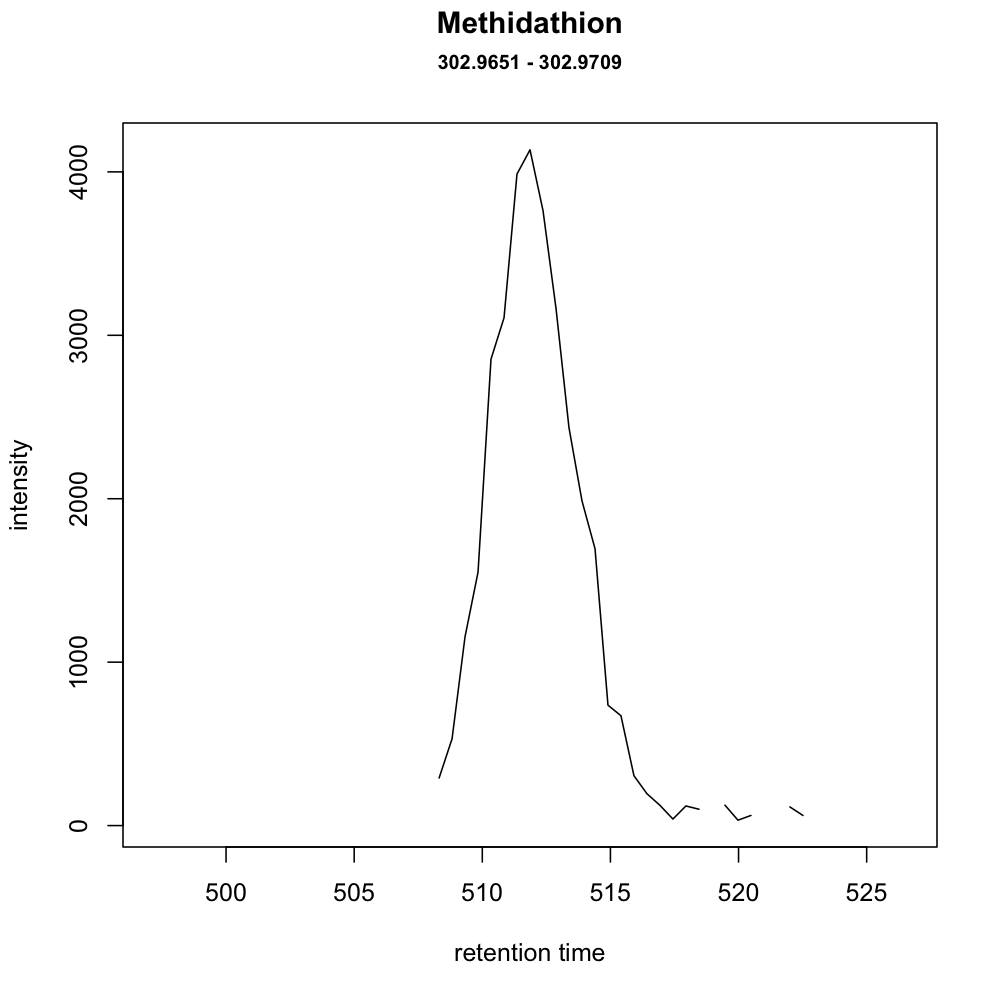

Supplement: Supplementary file 3 — (ZIP 2279 kb) [file 216_2018_1028_MOESM3_ESM.zip › EIC_Methidathion.png]

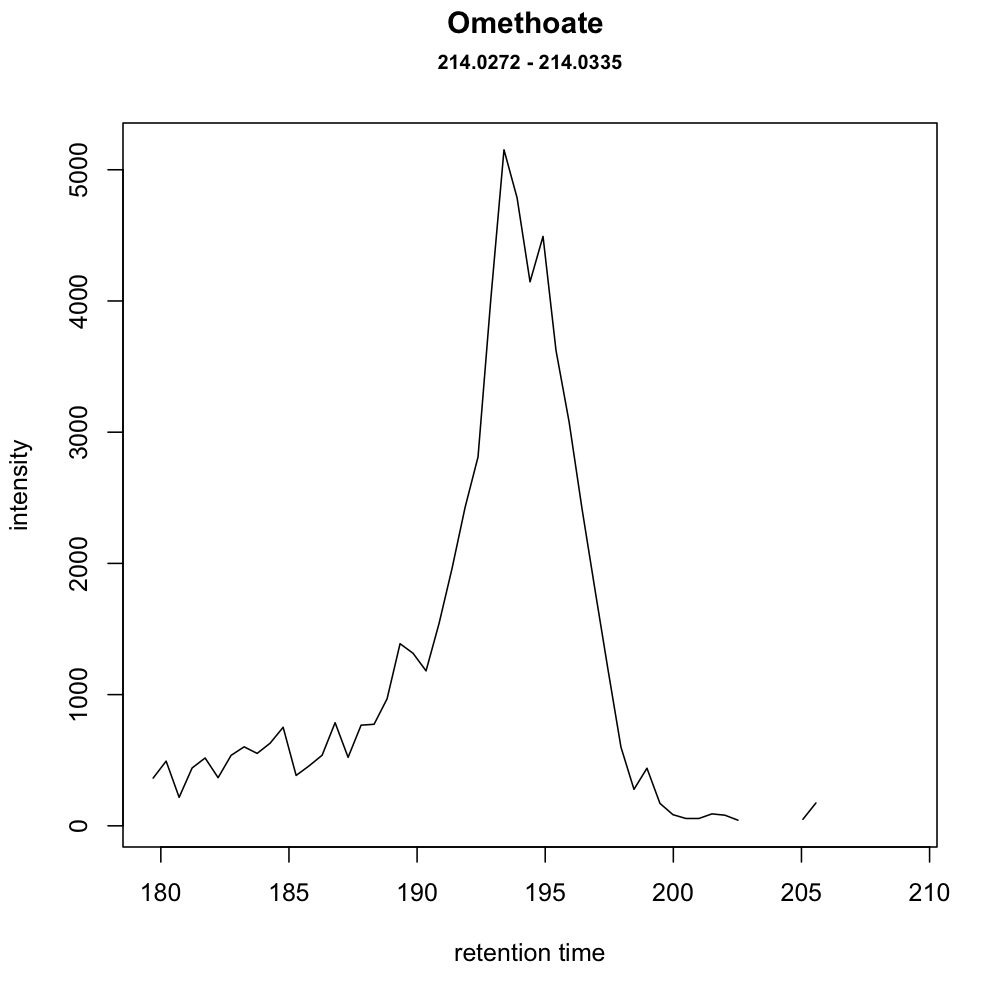

Supplement: Supplementary file 3 — (ZIP 2279 kb) [file 216_2018_1028_MOESM3_ESM.zip › EIC_Omethoate .png]

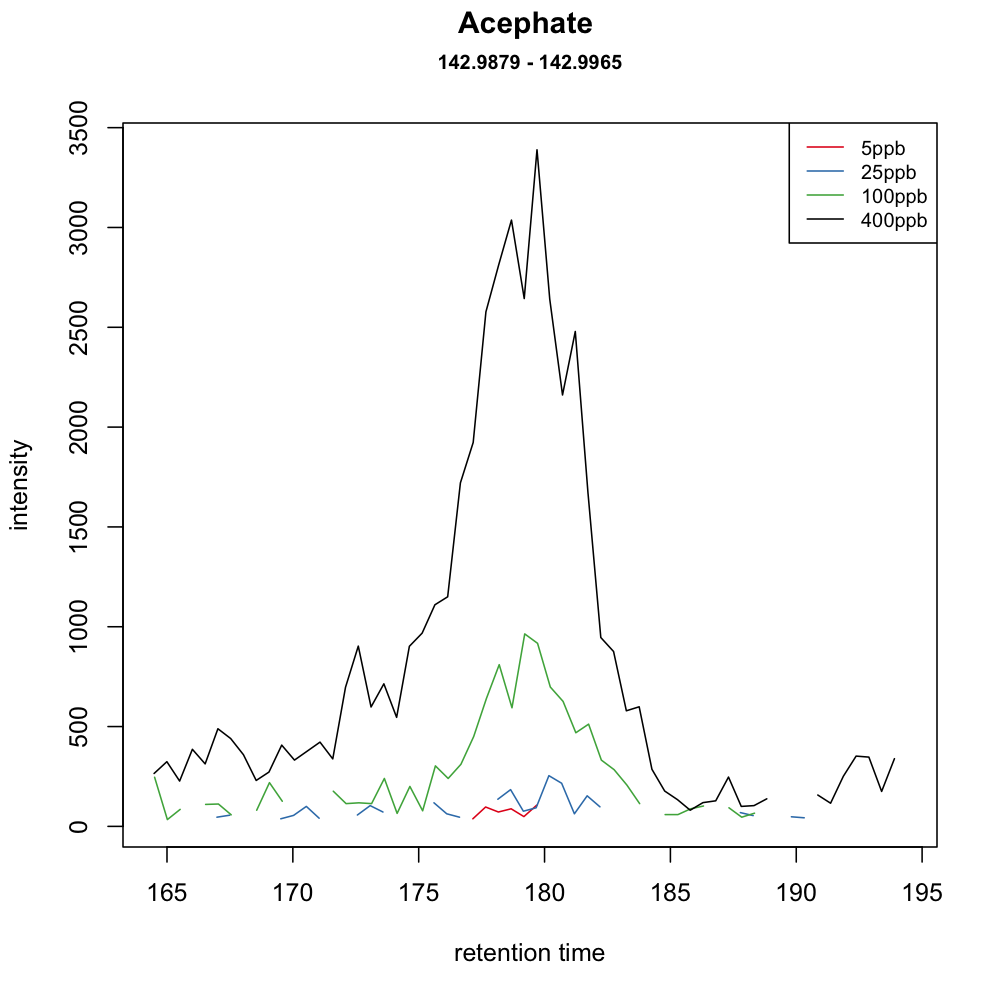

Supplement: Supplementary file 3 — (ZIP 2279 kb) [file 216_2018_1028_MOESM3_ESM.zip › EIC_overlayAcephate.png]

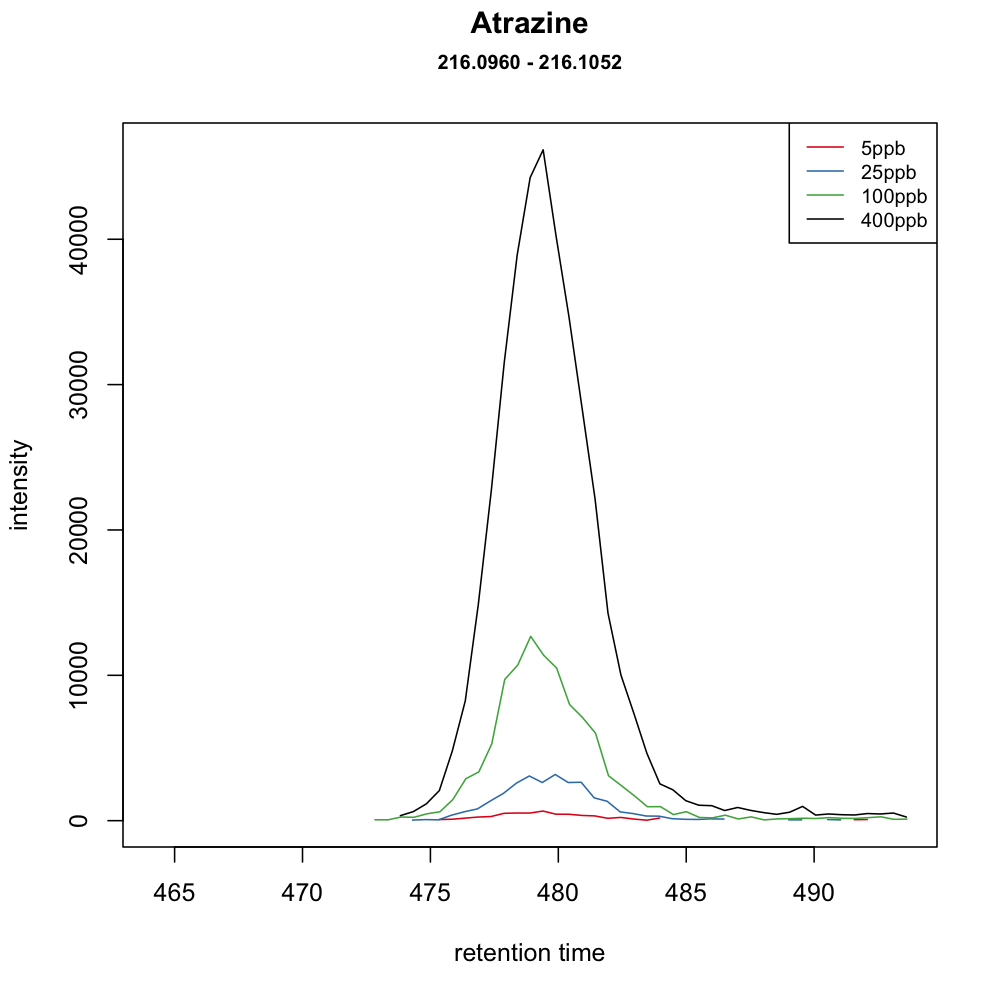

Supplement: Supplementary file 3 — (ZIP 2279 kb) [file 216_2018_1028_MOESM3_ESM.zip › EIC_overlayAtrazine.png]

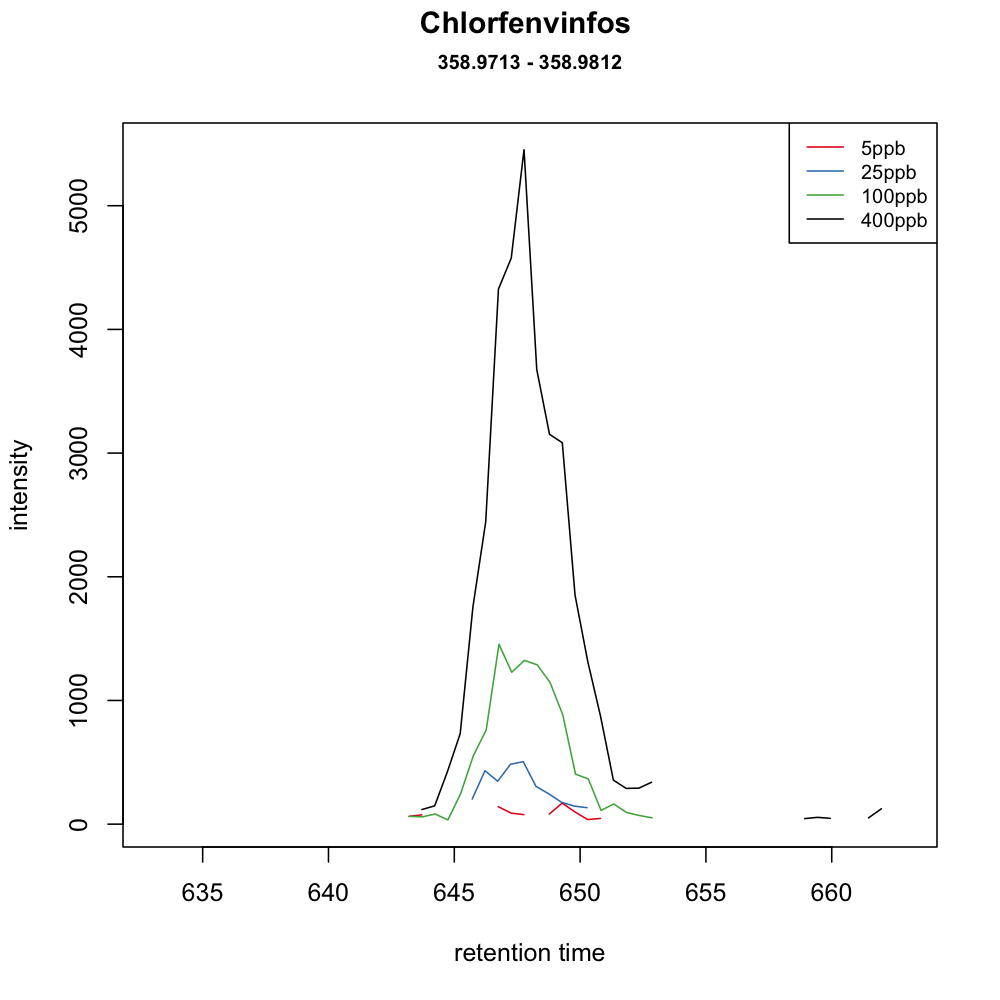

Supplement: Supplementary file 3 — (ZIP 2279 kb) [file 216_2018_1028_MOESM3_ESM.zip › EIC_overlayChlorfenvinfos .png]

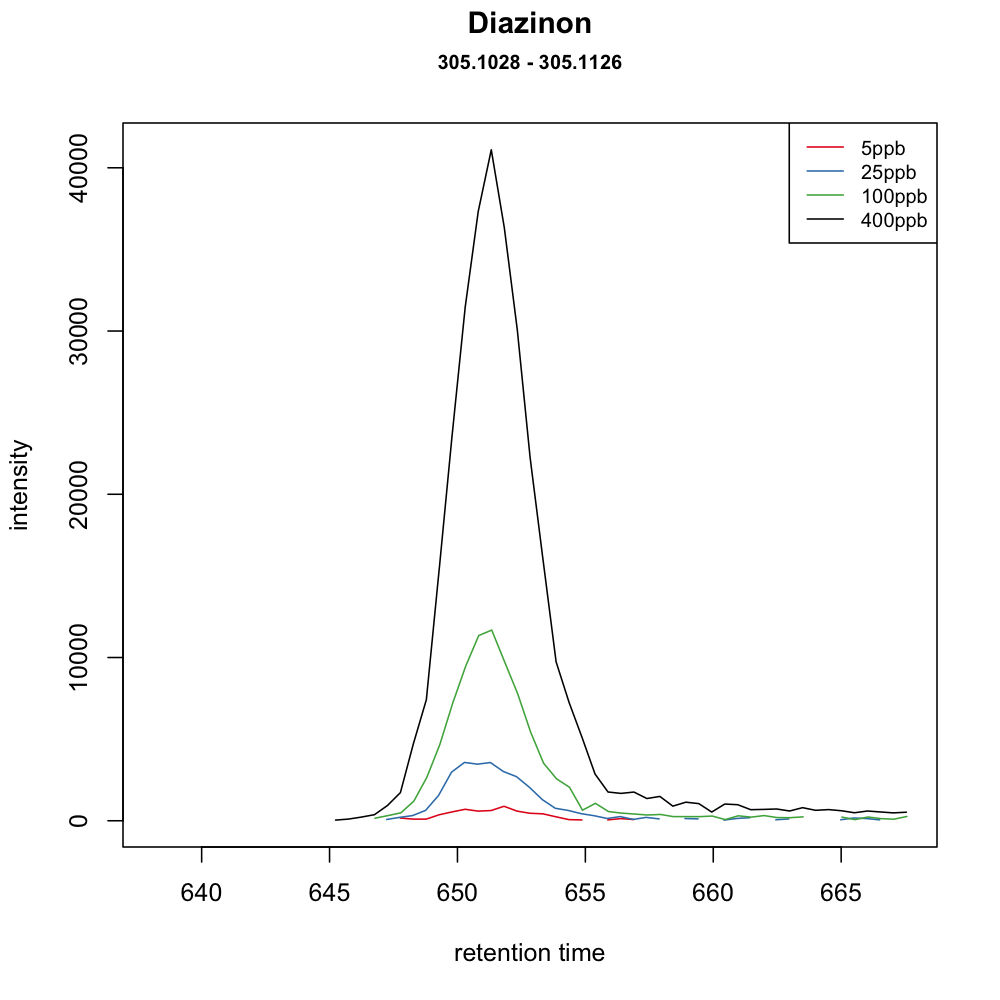

Supplement: Supplementary file 3 — (ZIP 2279 kb) [file 216_2018_1028_MOESM3_ESM.zip › EIC_overlayDiazinon.png]

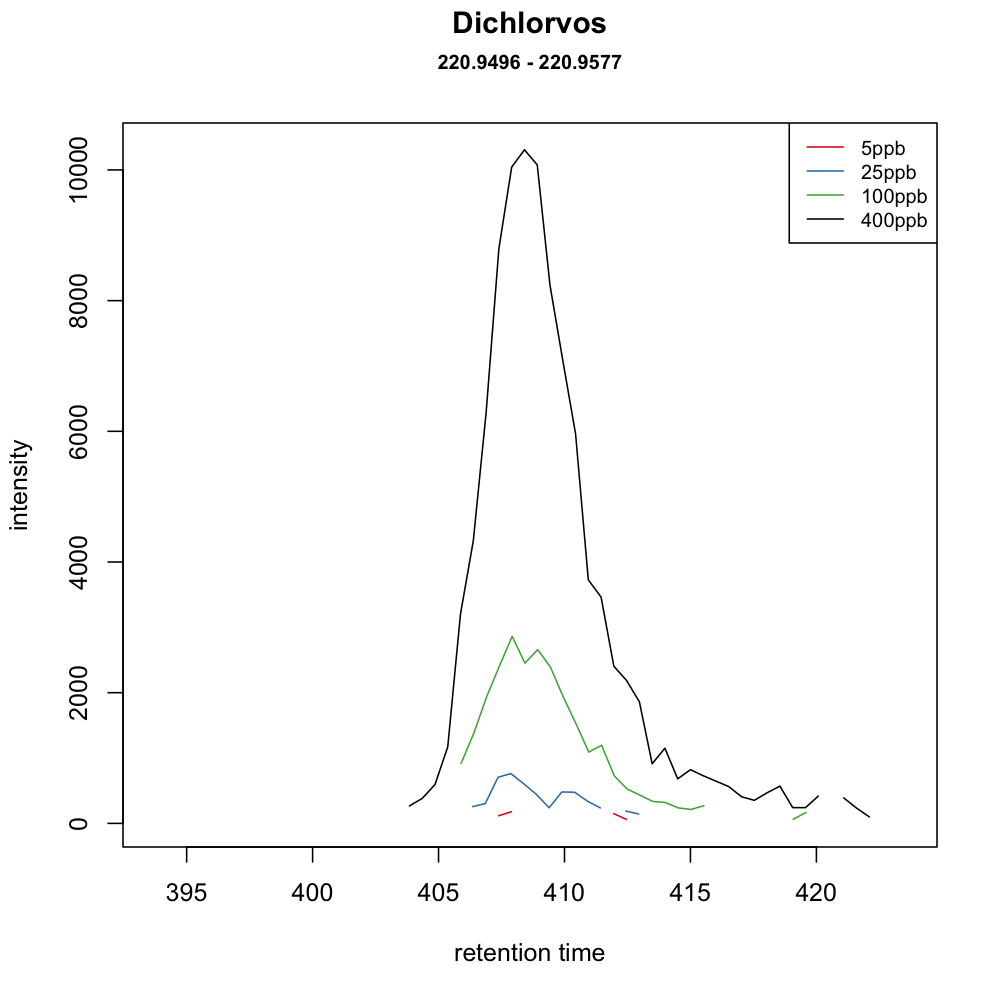

Supplement: Supplementary file 3 — (ZIP 2279 kb) [file 216_2018_1028_MOESM3_ESM.zip › EIC_overlayDichlorvos.png]

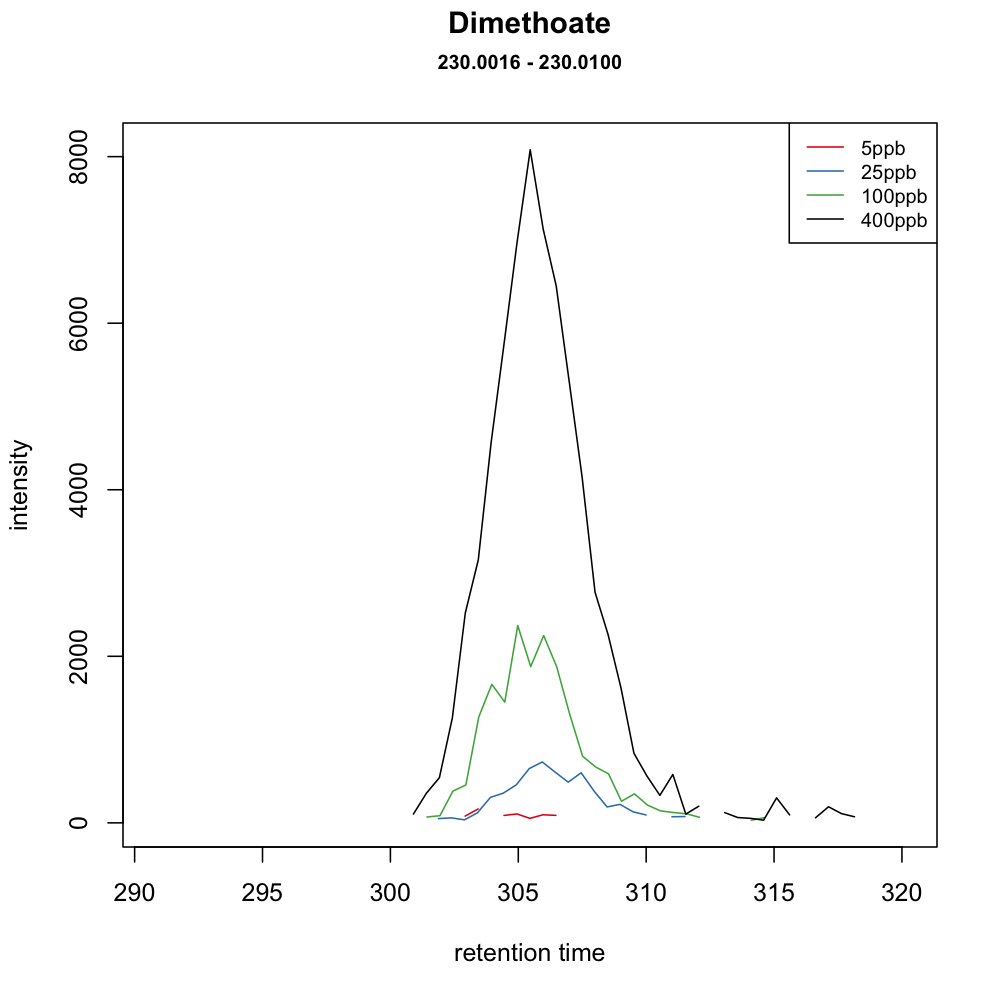

Supplement: Supplementary file 3 — (ZIP 2279 kb) [file 216_2018_1028_MOESM3_ESM.zip › EIC_overlayDimethoate.png]

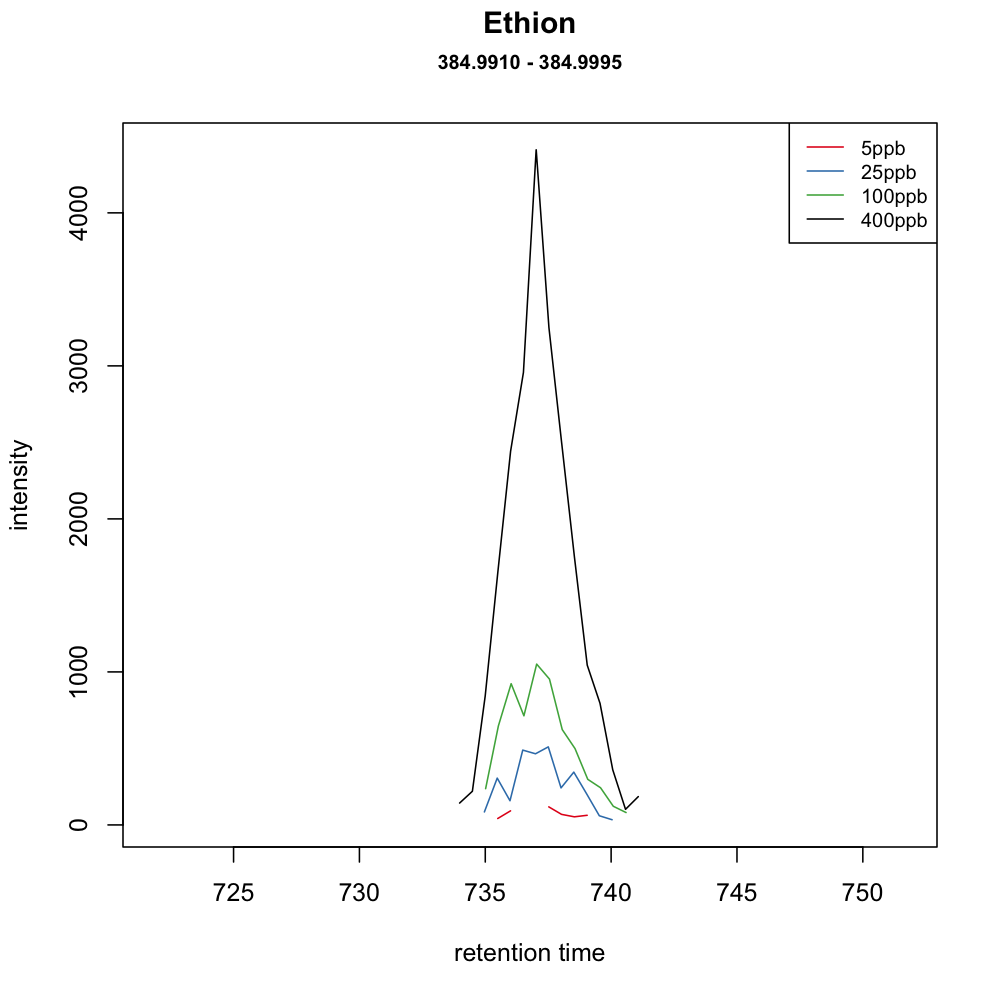

Supplement: Supplementary file 3 — (ZIP 2279 kb) [file 216_2018_1028_MOESM3_ESM.zip › EIC_overlayEthion.png]

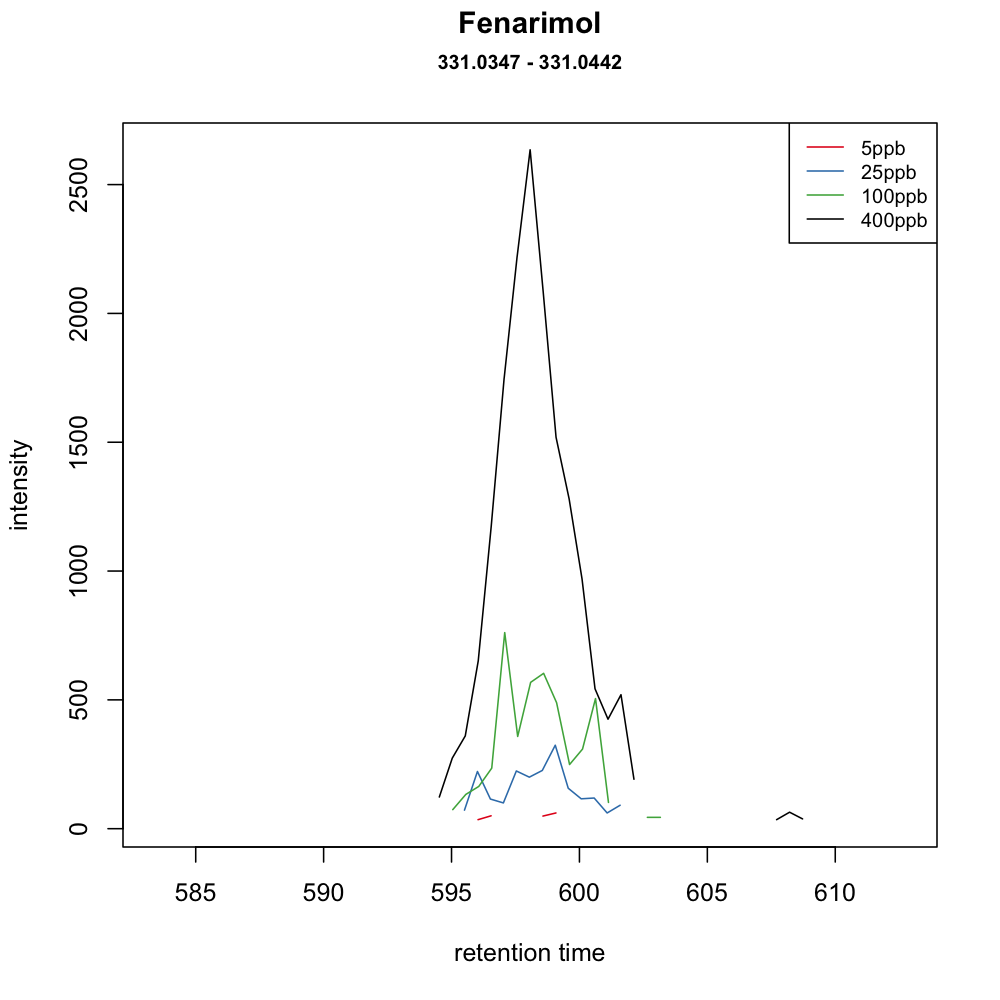

Supplement: Supplementary file 3 — (ZIP 2279 kb) [file 216_2018_1028_MOESM3_ESM.zip › EIC_overlayFenarimol.png]

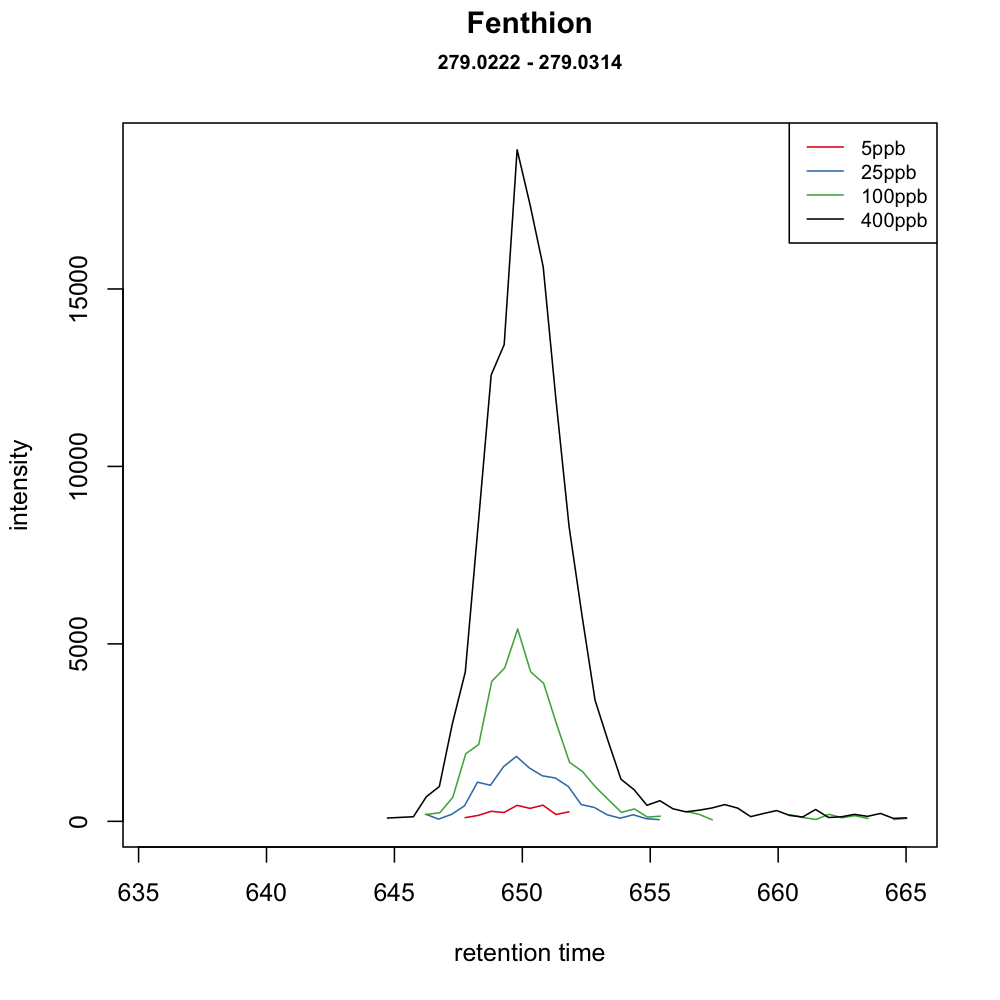

Supplement: Supplementary file 3 — (ZIP 2279 kb) [file 216_2018_1028_MOESM3_ESM.zip › EIC_overlayFenthion.png]

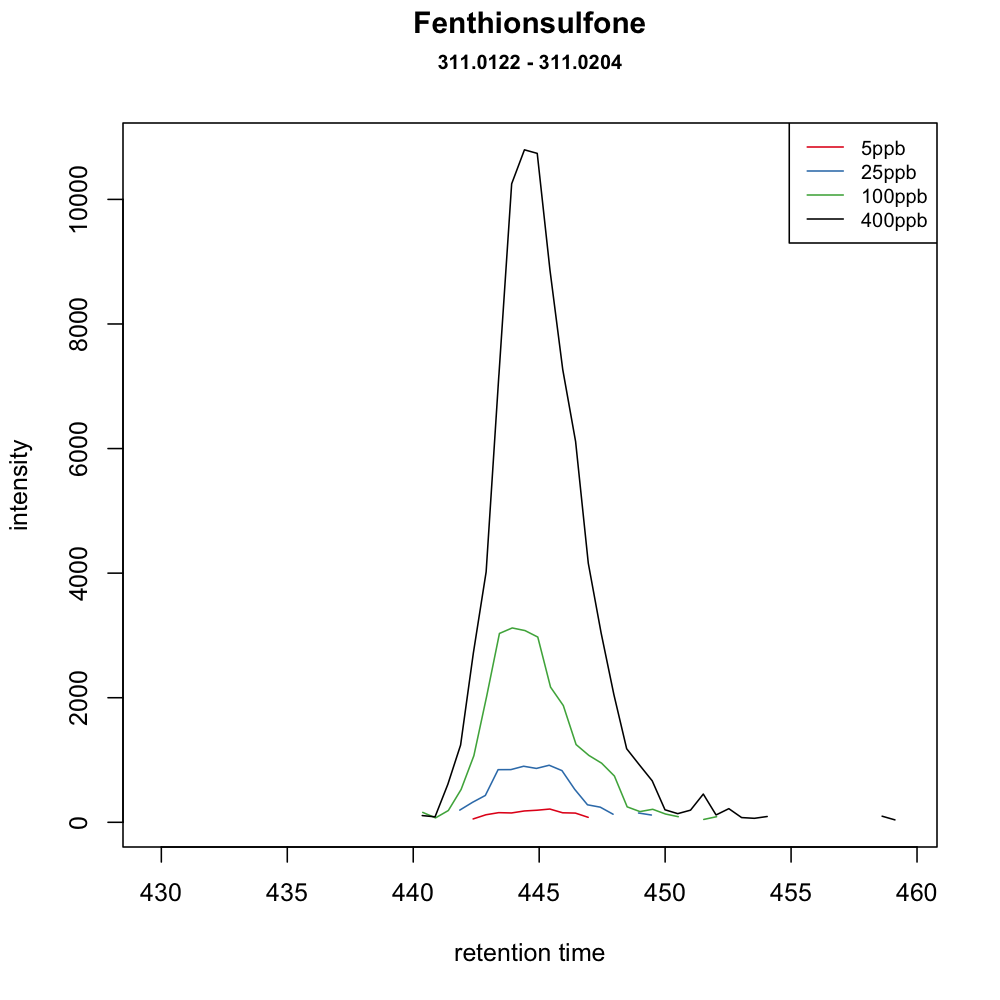

Supplement: Supplementary file 3 — (ZIP 2279 kb) [file 216_2018_1028_MOESM3_ESM.zip › EIC_overlayFenthionsulfone.png]

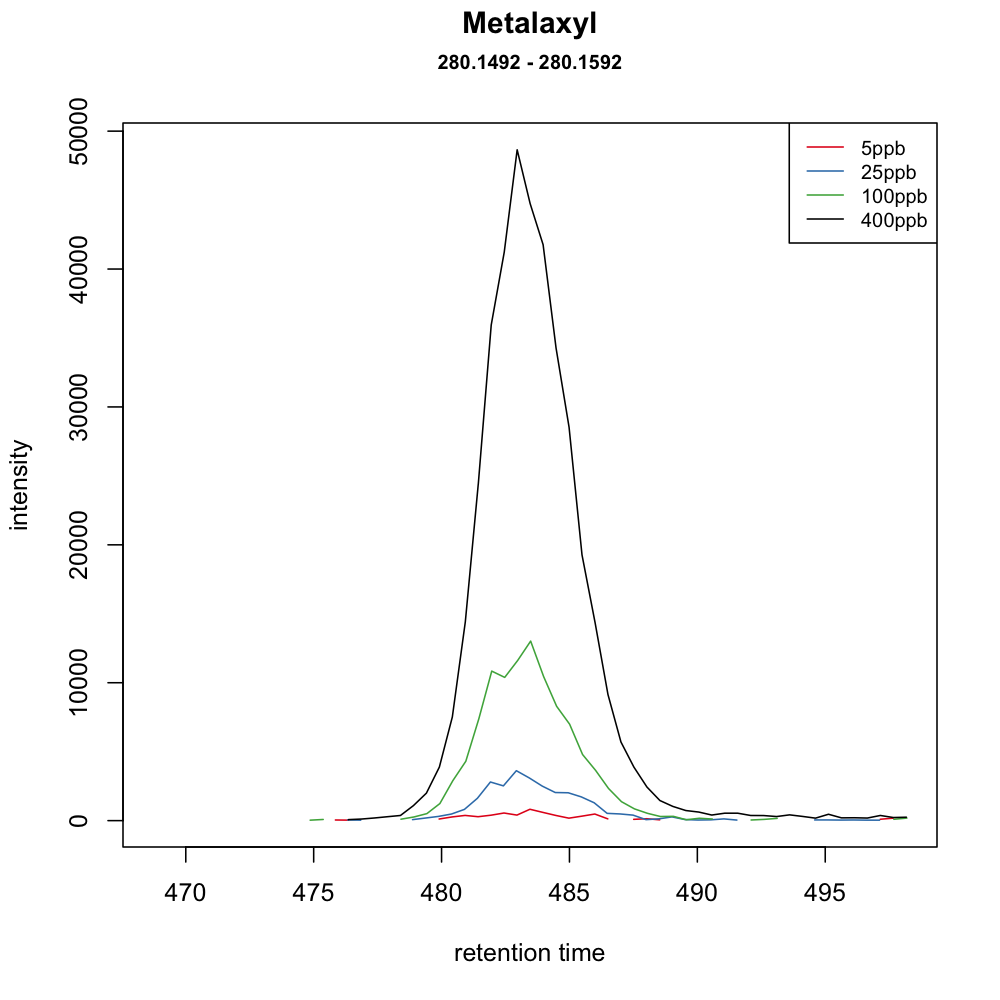

Supplement: Supplementary file 3 — (ZIP 2279 kb) [file 216_2018_1028_MOESM3_ESM.zip › EIC_overlayMetalaxyl.png]

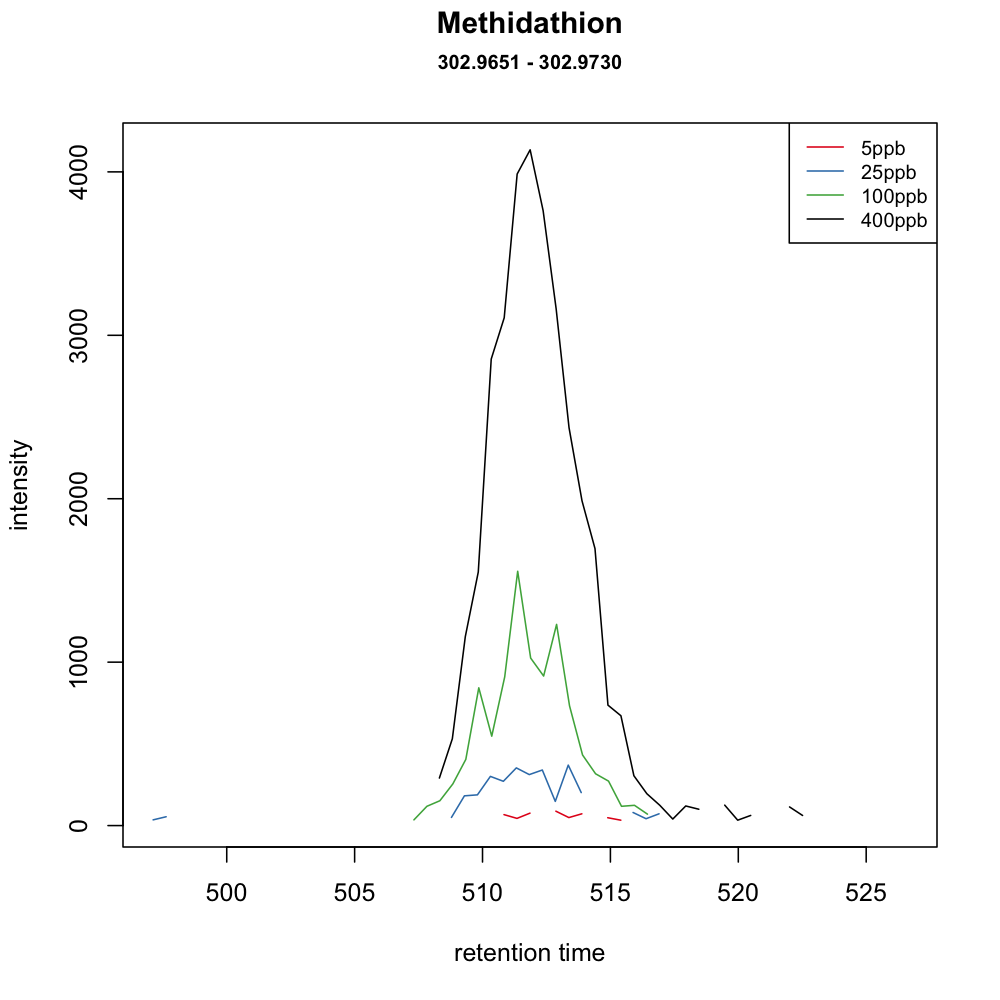

Supplement: Supplementary file 3 — (ZIP 2279 kb) [file 216_2018_1028_MOESM3_ESM.zip › EIC_overlayMethidathion.png]

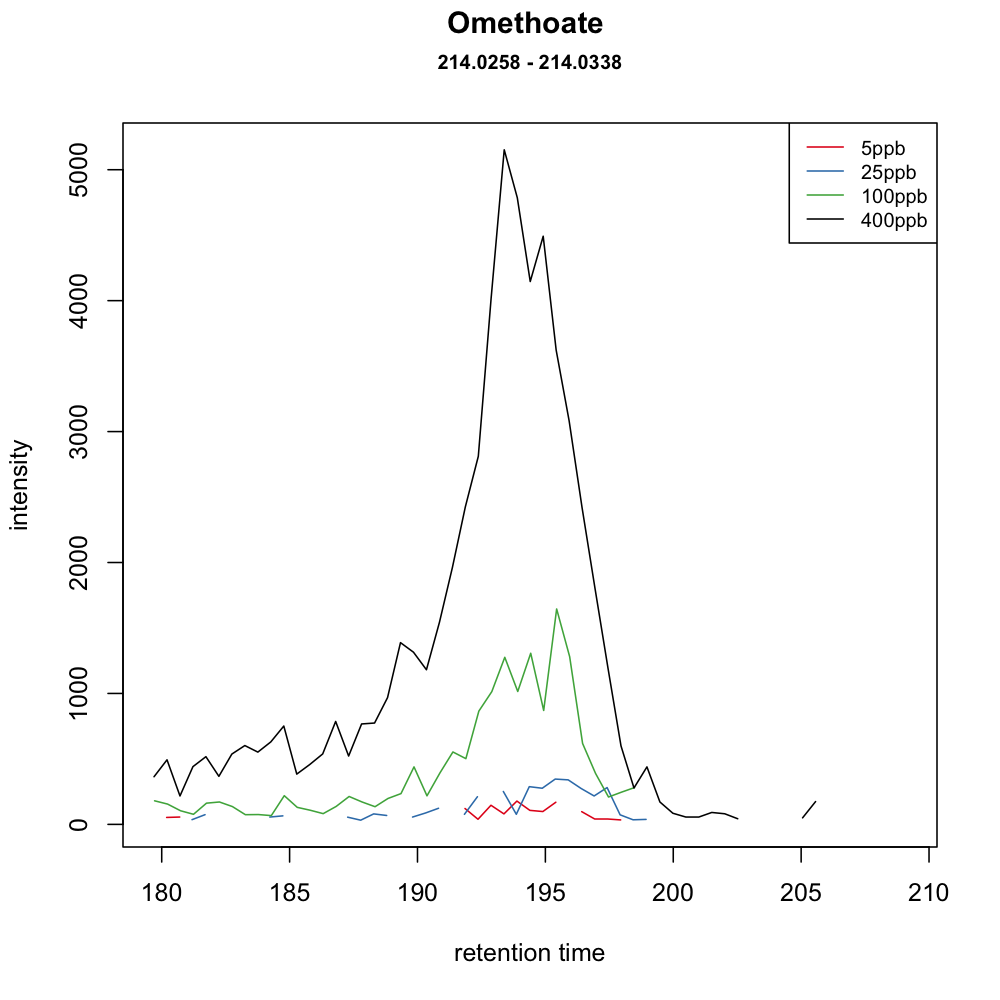

Supplement: Supplementary file 3 — (ZIP 2279 kb) [file 216_2018_1028_MOESM3_ESM.zip › EIC_overlayOmethoate .png]

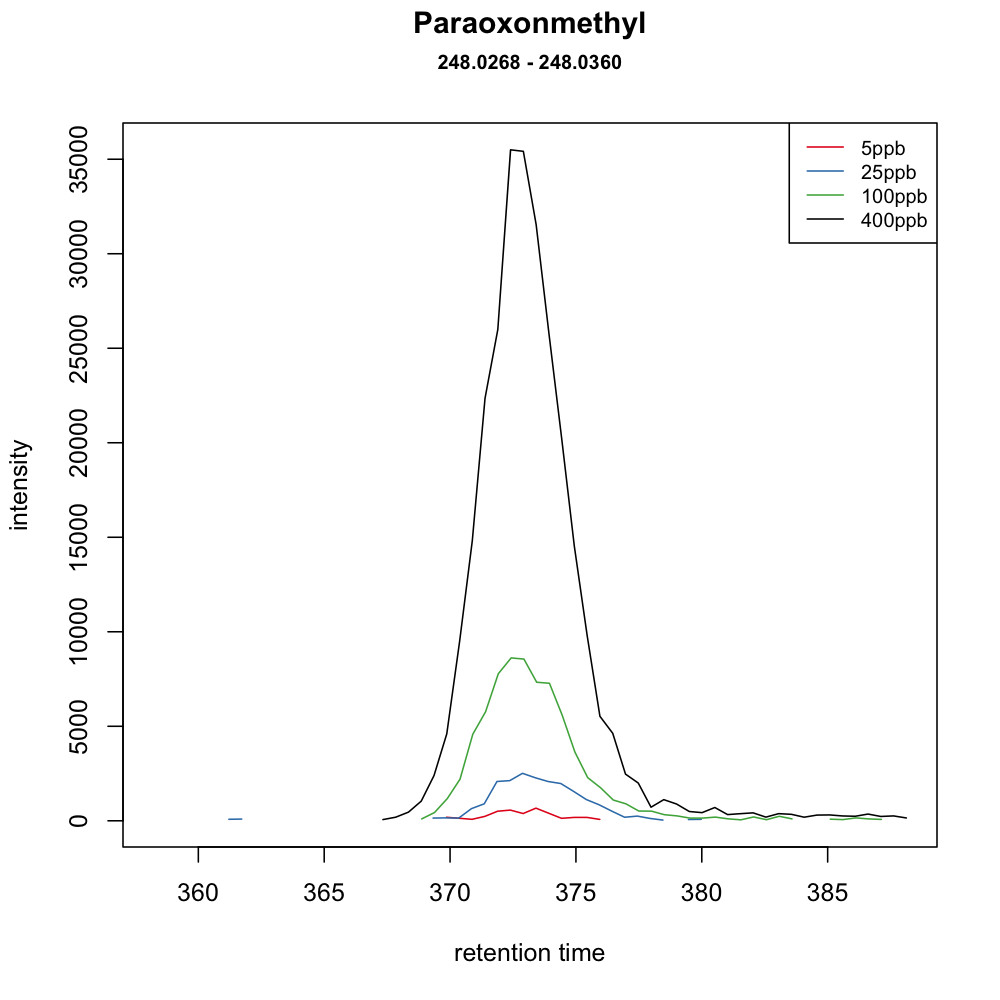

Supplement: Supplementary file 3 — (ZIP 2279 kb) [file 216_2018_1028_MOESM3_ESM.zip › EIC_overlayParaoxonmethyl.png]

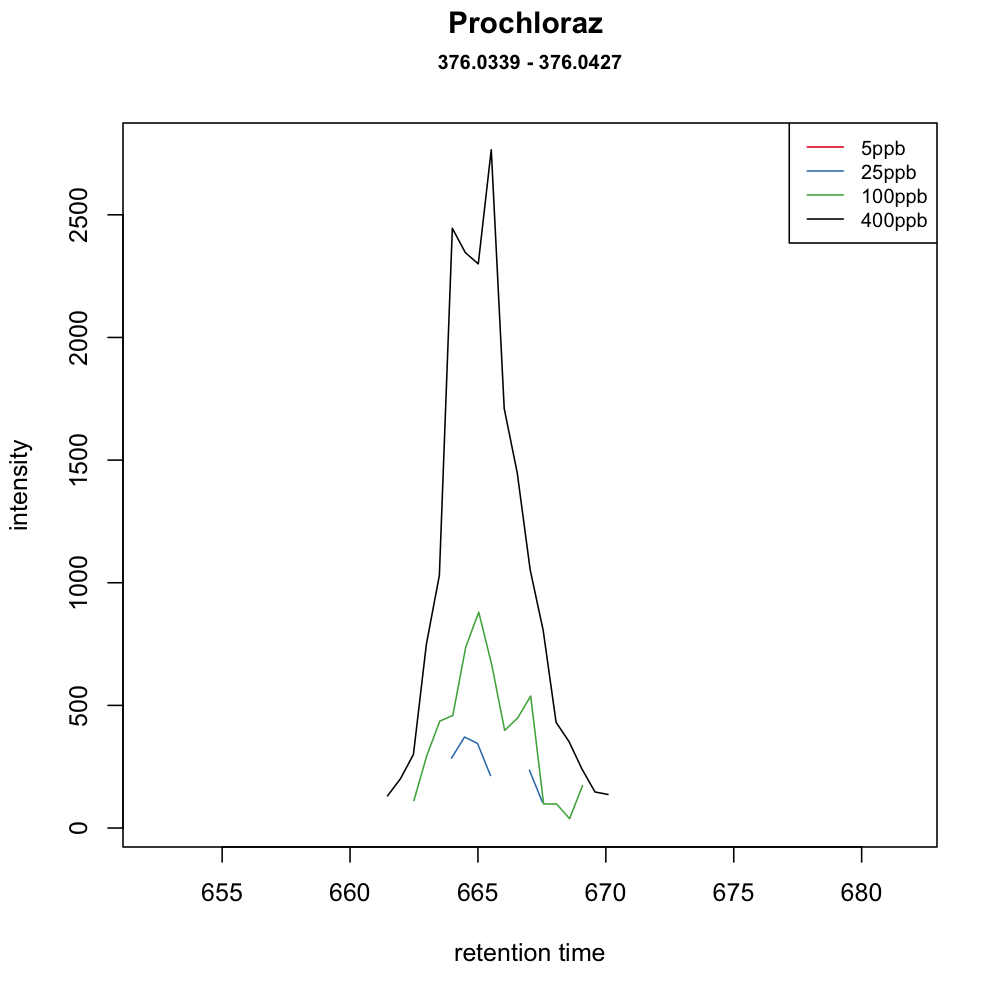

Supplement: Supplementary file 3 — (ZIP 2279 kb) [file 216_2018_1028_MOESM3_ESM.zip › EIC_overlayProchloraz .png]

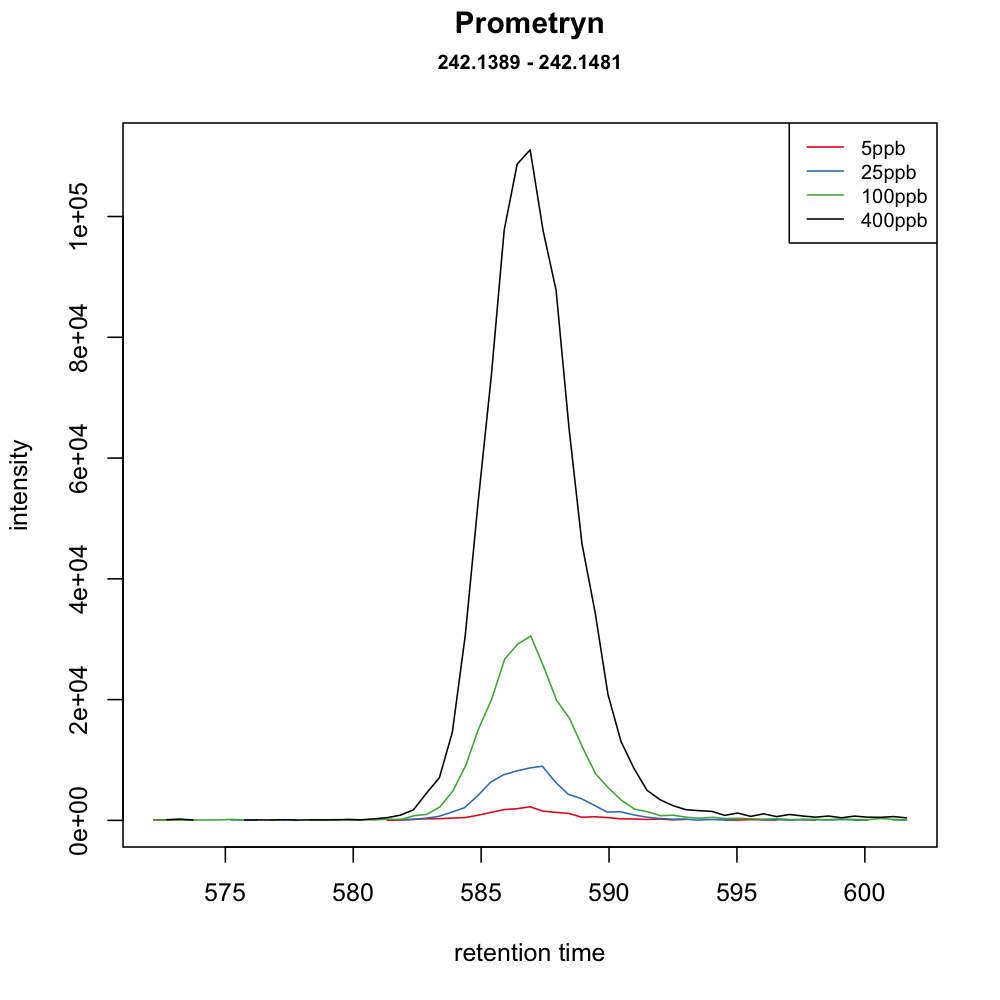

Supplement: Supplementary file 3 — (ZIP 2279 kb) [file 216_2018_1028_MOESM3_ESM.zip › EIC_overlayPrometryn.png]

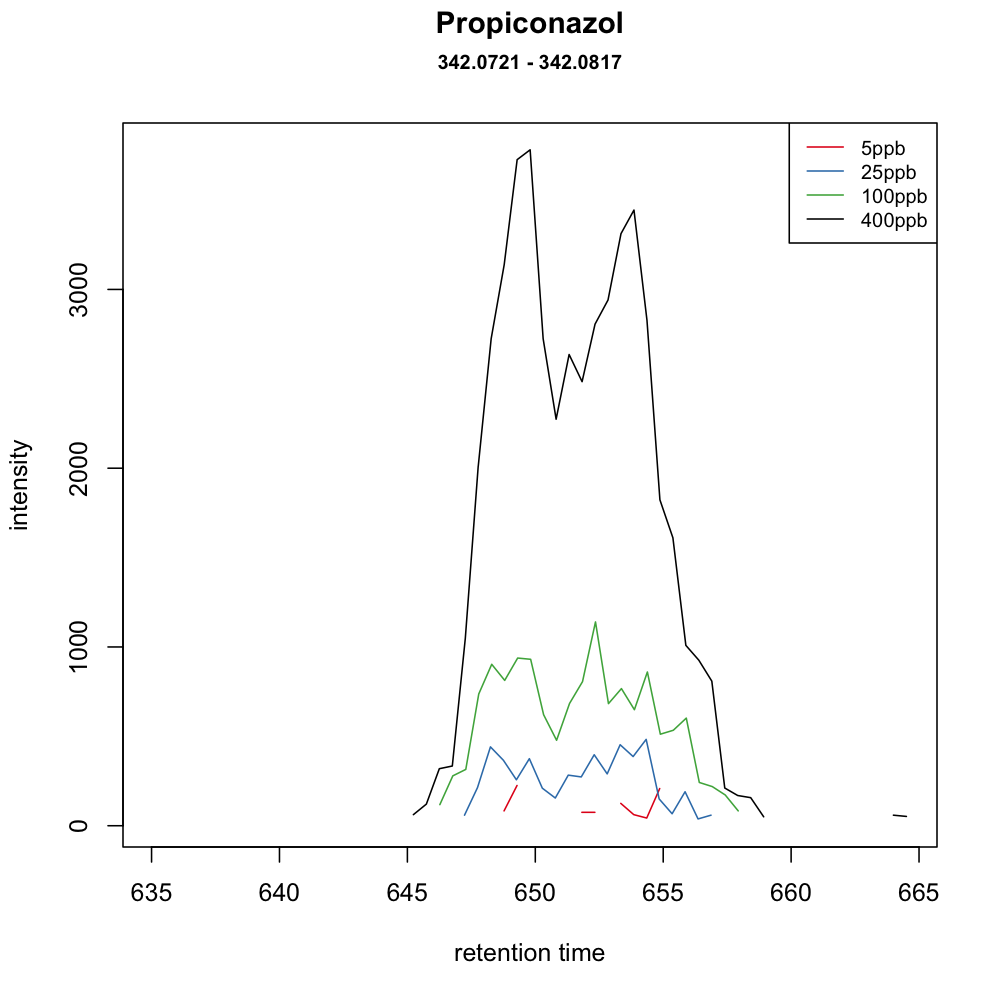

Supplement: Supplementary file 3 — (ZIP 2279 kb) [file 216_2018_1028_MOESM3_ESM.zip › EIC_overlayPropiconazol .png]

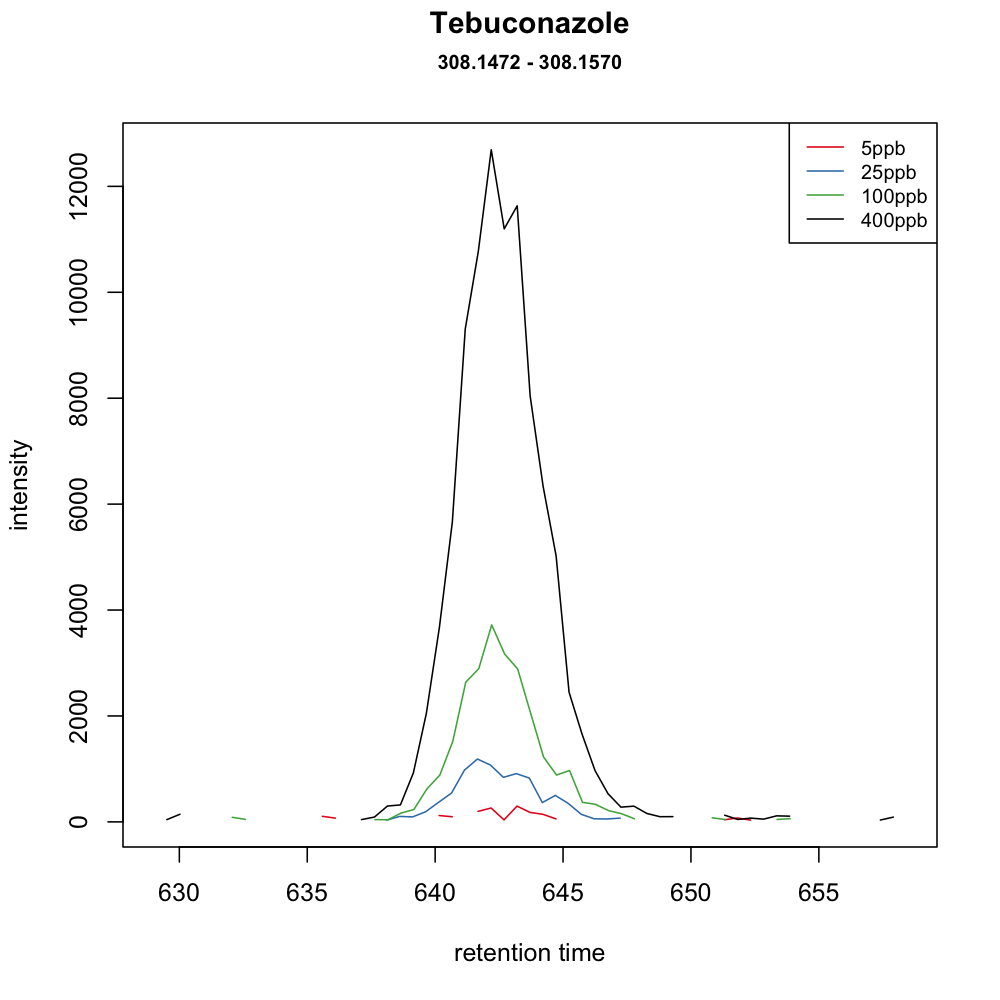

Supplement: Supplementary file 3 — (ZIP 2279 kb) [file 216_2018_1028_MOESM3_ESM.zip › EIC_overlayTebuconazole.png]

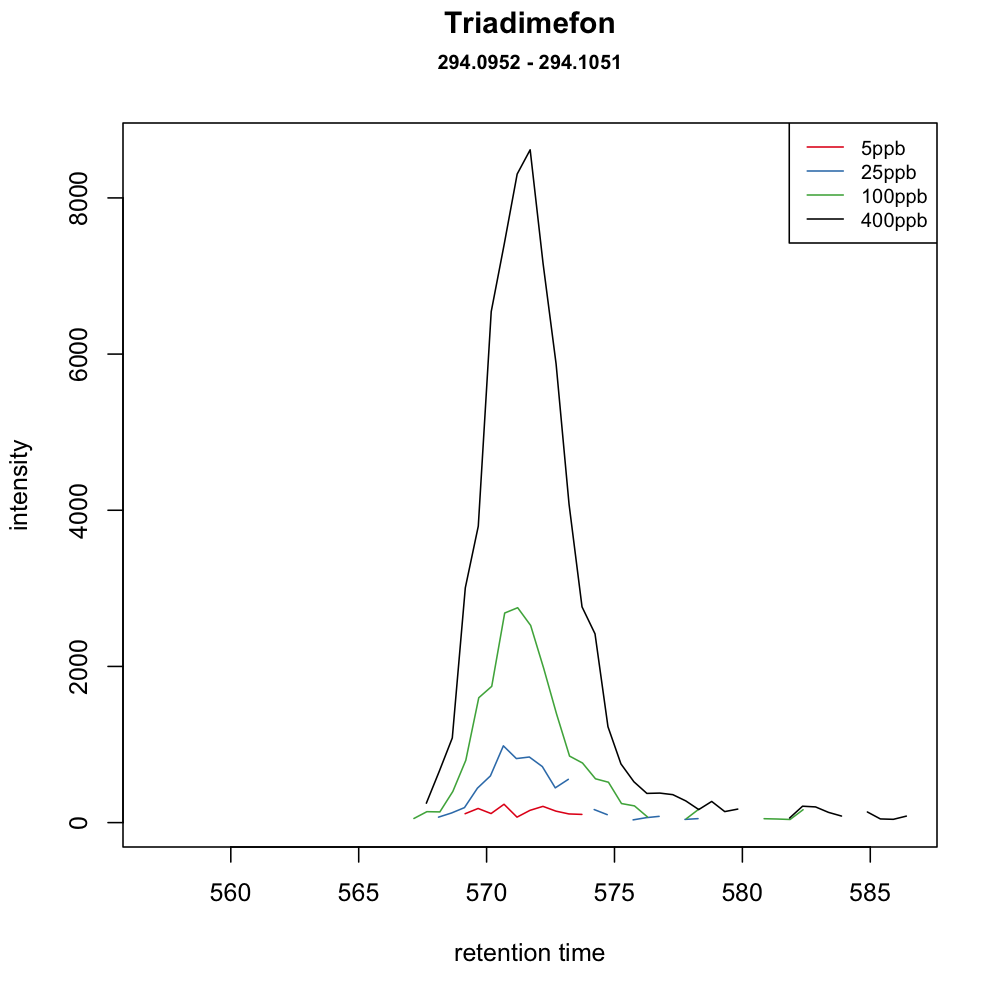

Supplement: Supplementary file 3 — (ZIP 2279 kb) [file 216_2018_1028_MOESM3_ESM.zip › EIC_overlayTriadimefon.png]

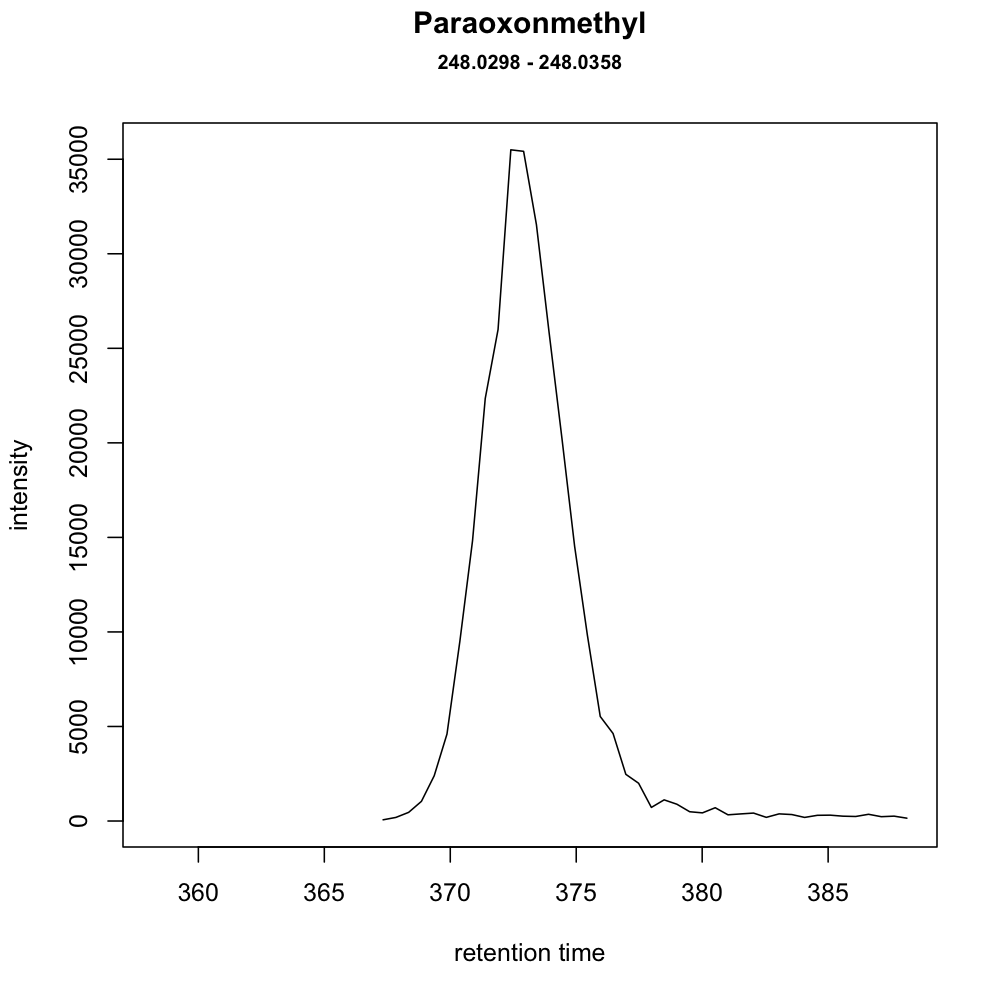

Supplement: Supplementary file 3 — (ZIP 2279 kb) [file 216_2018_1028_MOESM3_ESM.zip › EIC_Paraoxonmethyl.png]

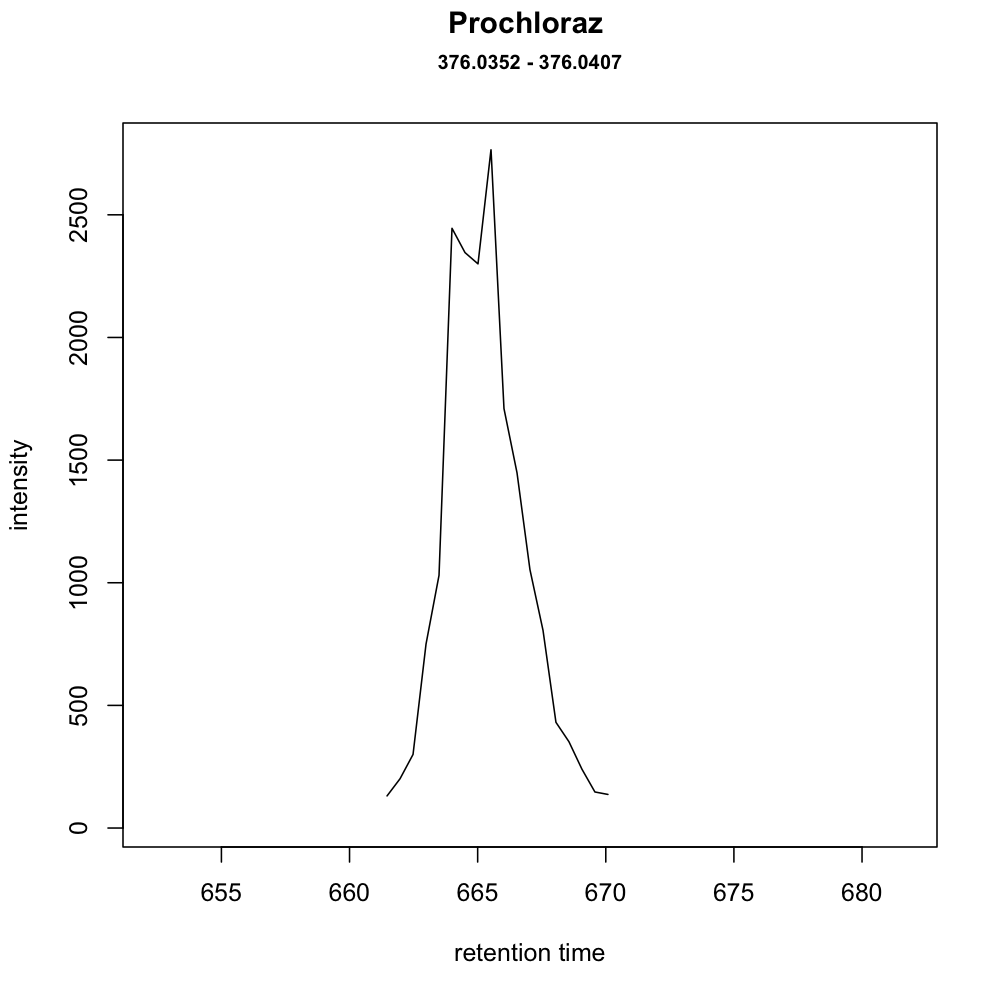

Supplement: Supplementary file 3 — (ZIP 2279 kb) [file 216_2018_1028_MOESM3_ESM.zip › EIC_Prochloraz .png]

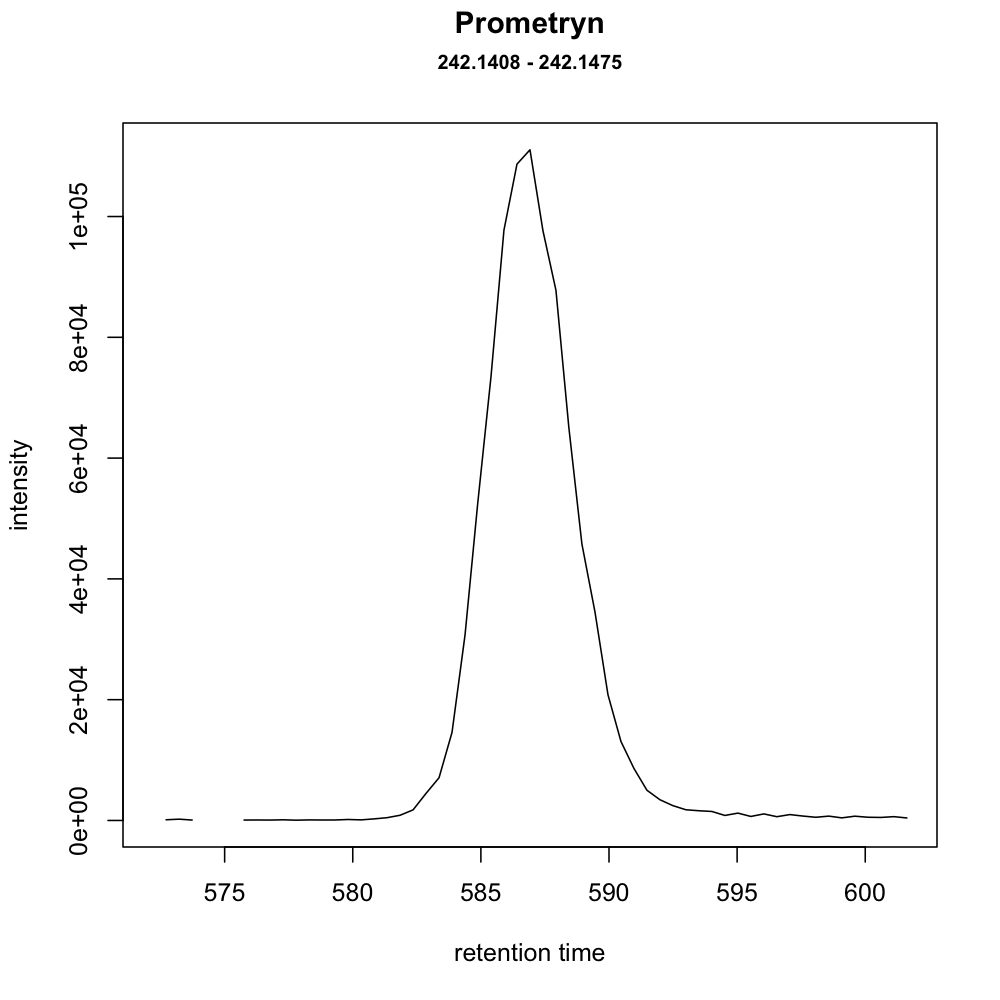

Supplement: Supplementary file 3 — (ZIP 2279 kb) [file 216_2018_1028_MOESM3_ESM.zip › EIC_Prometryn.png]

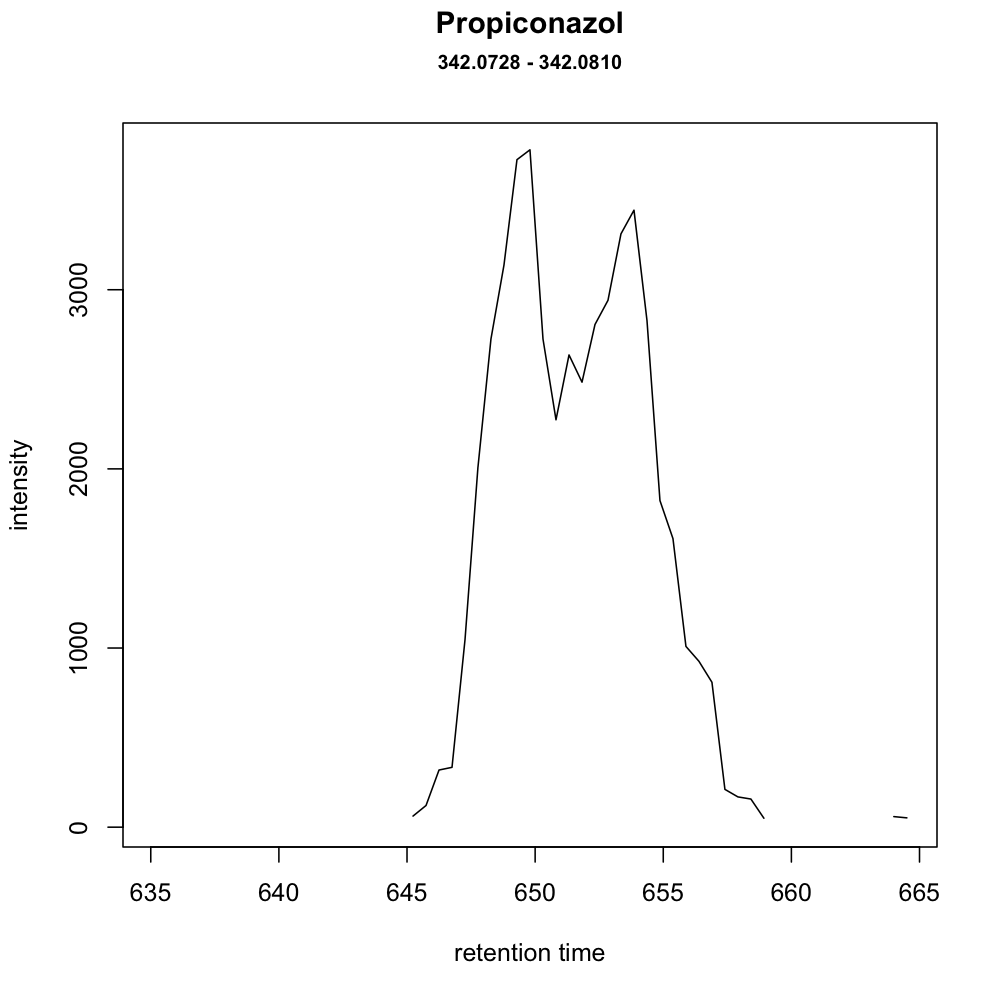

Supplement: Supplementary file 3 — (ZIP 2279 kb) [file 216_2018_1028_MOESM3_ESM.zip › EIC_Propiconazol .png]

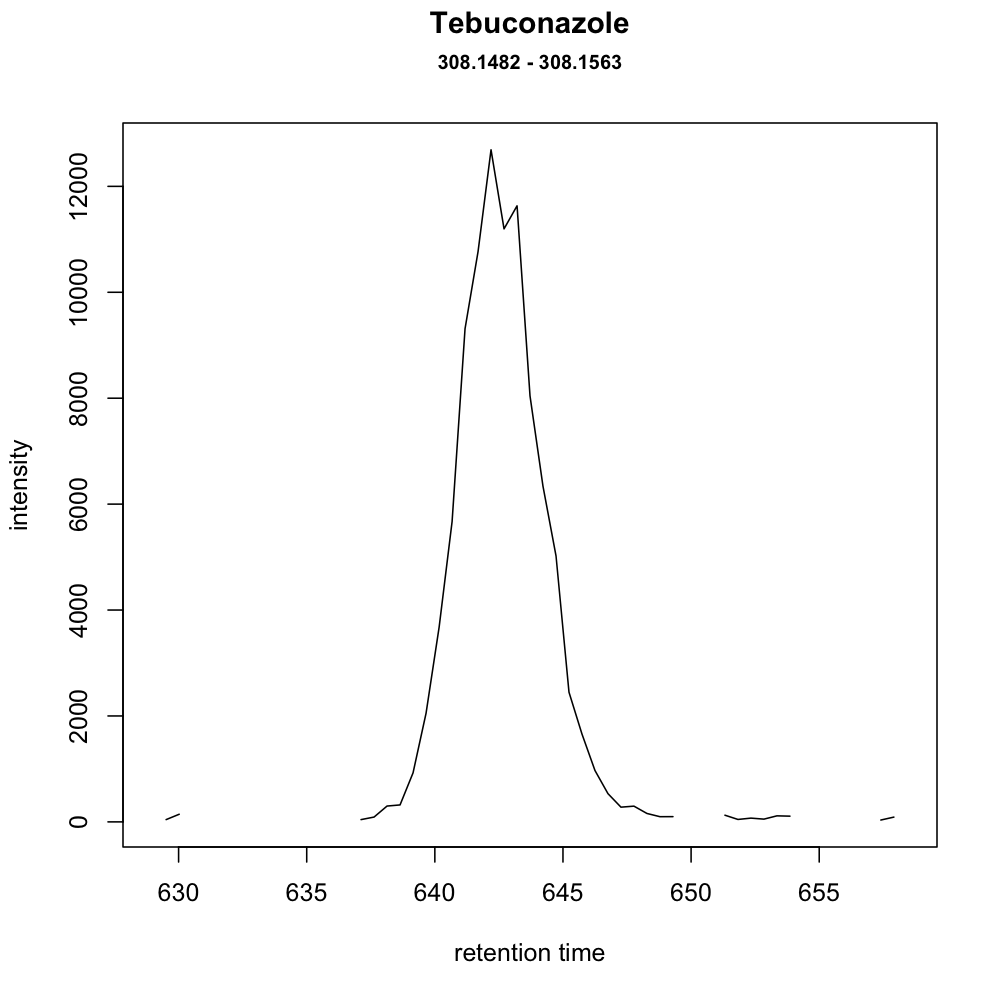

Supplement: Supplementary file 3 — (ZIP 2279 kb) [file 216_2018_1028_MOESM3_ESM.zip › EIC_Tebuconazole.png]

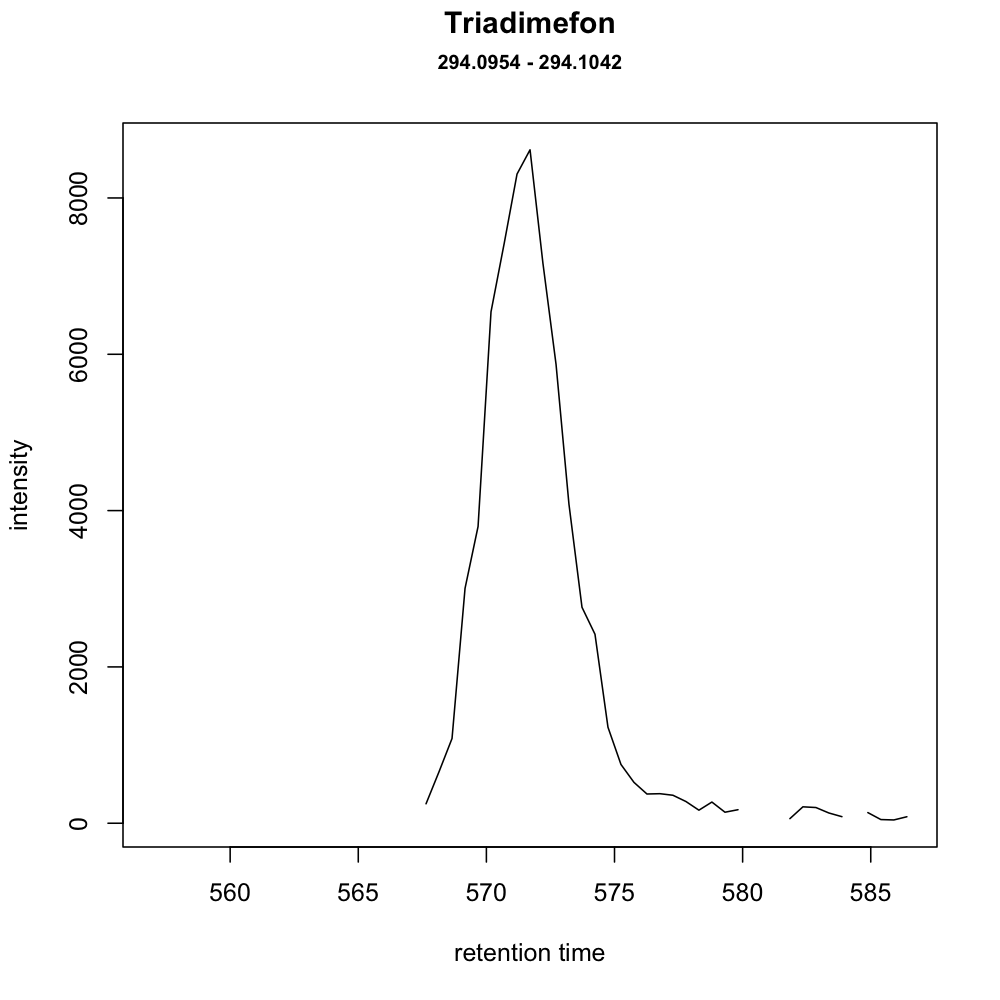

Supplement: Supplementary file 3 — (ZIP 2279 kb) [file 216_2018_1028_MOESM3_ESM.zip › EIC_Triadimefon.png]

## Slide 1
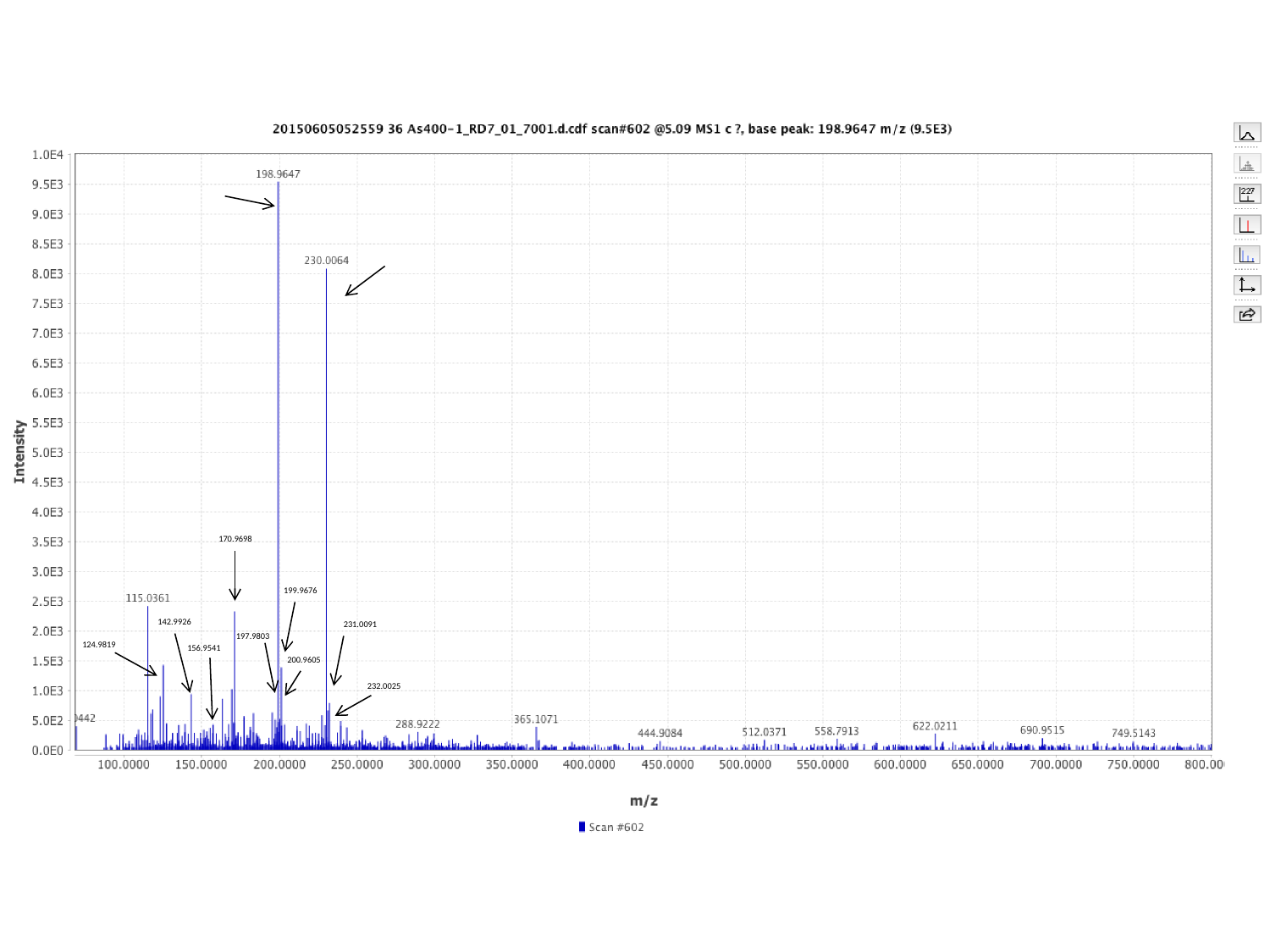

#
170.9698
199.9676
142.9926
231.0091
197.9803
124.9819
156.9541
200.9605
232.0025

Supplement: Supplementary file 4 — (PPTX 170 kb) [file 216_2018_1028_MOESM4_ESM.pptx]
